# Millimeters long super flexible Mn_5_Si_3_@SiO_2_ electrical nanocables applicable in harsh environments

**DOI:** 10.1038/s41467-019-14244-5

**Published:** 2020-01-31

**Authors:** Yong Sun, Bo Sun, Jingbo He, Guowei Yang, Chengxin Wang

**Affiliations:** State Key Laboratory of Optoelectronic Materials and Technologies, School of Materials Science and Engineering, Sun Yat-sen (Zhongshan) University, Guangzhou, 510275 People’s Republic of China

**Keywords:** Nanoscale materials, Nanowires, Structural properties

## Abstract

Providing high performance electrical nano-interconnects for micro-nano electronics that are robust in harsh environments is highly demanded. Today, electrical nano-interconnects based on metallic nanowires, e.g. Ag and Cu, are limited by their positive physicochemical reactivity and ductility under large strain (i.e. irreversible dislocations and local necking-down elongation) at high temperatures or in strong oxidizing and acidic environments. Herein, to overcome these limitations, high-quality millimetre-sized soft manganese-based silicide (Mn_5_Si_3_@SiO_2_) nanowire nanocables are designed via a glassy Si–Mn–O matrix assisted growth. The proposed nanocables exhibit good electrical performance (resistivity of 1.28 to 3.84×10^-6^ Ωm and maximum current density 1.22 to 3.54×10^7^ A cm^−2^) at temperatures higher than 317°C in air atmosphere, strongly acidic (HCl, PH=1.0) and oxidizing (H_2_O_2_, 10%) ambient, and under complex electric field. The proposed Mn_5_Si_3_@SiO_2_ nanocables, which withstand a strain of 16.7% free of failure, could be exploited for diverse applications in flexible electronics and complex wiring configurations.

## Abstract (web summary)

Though high performance electrical interconnects are required for micro/nano electronics, existing metallic nanowires lack structural and electrical stability. Here, the authors report soft manganese-based silicide nanowires with high electrical and structural performance in harsh environments.

## Introduction

Nanoscopic electrical interconnect is a key link in micro-nano electronics, optoelectronics, bioelectronics and also complex integrated functional nanosystem^1–3^. As natural electron transport channels, metallic nanowires are frequently investigated as candidates to construct an electrical circuit, for which compatibility to the device fabrication technology and also the working environment must be simultaneously assessed^4,5^. For example, in special applications, the devices frequently work on extreme conditions, where the structural stability and electrical availability of the components may be challenged. Except for the basic electrical figure of merits, i.e. conductivity and maximum current density, the resistance to harsh surroundings accordingly deserve to be considered as expanded requirement^6,7^. In single nanowires based electrical interconnect, several possible difficulties should be mentioned, including surface interaction with chemical species in chemical and biological environments, interferences due to current leakage, structural damage at elevated temperature in air atmosphere due to lattice failure and accumulated oxidation event, and also irreversible plastic deformation like local necking-down elongation and dense dislocations, especially in nanowires with low yield strength^8,9^. All of these would result in a sudden failure or accumulated electrical degeneration of the nanowire.

Metallic nanowires, such as Ag and Cu NWs, possess high conductivity and withstand very large current density. Amounts of interlaced nanowires can serve as planar electrodes applied in solar cell^10,11^, touch panel^12^, biological sensor^7,13^, and so on^14,15^. These progresses may open the avenue of flexible, transparent, and portable electronics in post-ITO (Indium Tin Oxide) industry. Despite the highly developed fabrication technology and good performance, they behave deficient dealing with extreme conditions like elevated temperatures, oxidative and acidic environments. Electrical performance begin to degenerate in these nanowires below 200 °C^16^. Yu and colleague's group has reported that Cu NWs can be quickly oxidized by dissolved oxygen within several hours, which degenerate and evolve into a mace-like structure when stored in water or nonpolar organic solvents^17^. They are thusly not immediate compatible to high temperature, chemical and biological environments^7^.

Alloying silicide compounds also display potential as electrical interconnects benefiting from their metallic-like electron transport behavior^18–21^ and good compatibility to silicon based device^22–25^. Noticeably, the ceramic attribute endow them with outstanding physicochemical stability and mechanical performance like large elasticity modulus and high hardness^26^.

In the past, Mn_5_Si_3_ is widely investigated as an antiferromagnetic material^27–29^. Herein, a CVD growth of millimeter level Mn_5_Si_3_@SiO_2_ nanocables in molten glassy Si-Mn-O matrix was demonstrated here. With available integration of compacted SiO_2_ sheath and electrical Mn_5_Si_3_ core, such a configuration not only display considerable good electrical figure of merit (resistivity of 1.28–3.84 × 10^−6^ Ω m and maximum current density 1.22–3.54 × 10^7^ A cm^−2^), but great capability to deal with high-temperature (>317 °C), strong acidic, high oxidizing, complex electric field and current surroundings. Noticeably, with amorphous SiO_2_ shell dissipating considerable strain energy when bended, a single nanocable displays a mechanical flexibility, despite Mn_5_Si_3_ core is mechanically brittle and rigid. Even a ~16.7% bending strain loaded, there is no cracking event observed. The versatile mechanical behavior makes it competent to flexible electronics and also adjustable to complex wiring condition.

## Results

### Synthesis and characterizations of Mn_5_Si_3_@SiO_2_ nanocables

**Fig. 1:**
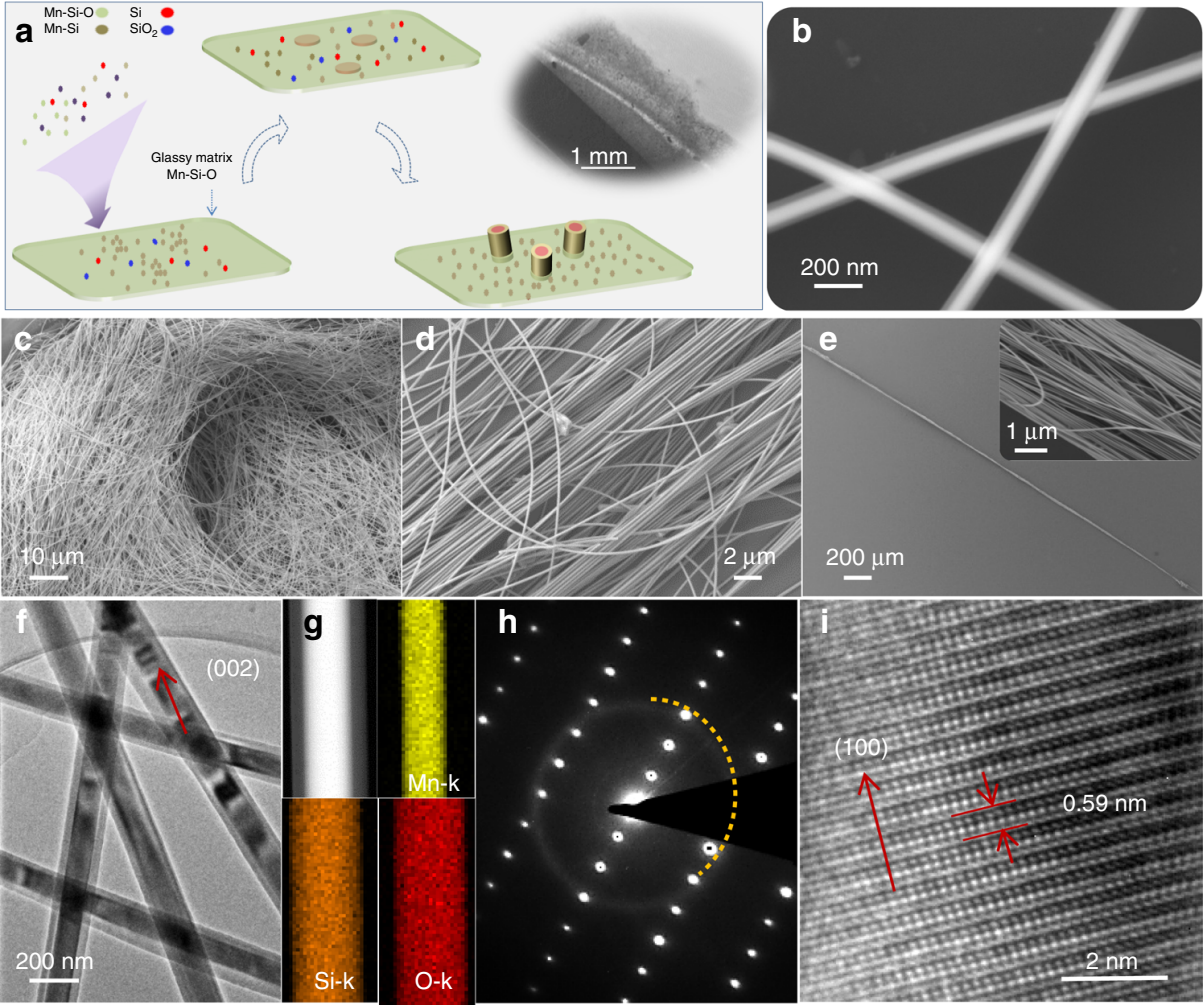
Growth illustration and characterization of Mn_5_Si_3_@SiO_2_ nanocables. **a** The growth schematic, inset shows the as-synthesized sample on ceramic sheet. **b** SEM observation of several core–shell nanowires. **c**–**d** Nanowire piles after being transferred to Si substrate. **e** A nanowire bundle composed of many quasi-orientated unites, inset shows the closer view. **f** Typical bright field TEM image of several core–shell nanowires. **g** The STEM-EDS mapping characterization of a piece of nanowire, which includes Mn, Si and O elements. **h** The SAED pattern of the nanowire, dash semi-circle denotes the signal corresponding to amorphous SiO_2_ sheath. **i** HRTEM image highlighting the nanowire growing along *c*-axial.

The sample was synthesized in a home-made horizontal tube furnace as previously demonstrated^30,31^. The growth of Mn_5_Si_3_ core–shell nanowires proceeded in a molten Mn-Si-O glassy matrix (Fig. 1a). In brief, a eutectic system was established by transporting dissociative source species like Mn-Si, Si, and Si-O into the pre-deposited Mn-Si-O matrix. Persistent source supplement and Mn_5_Si_3_ precipitation sustain a continuous growth of ultra-long nanowires (Supplementary Note 1). The resulted nanowires exhibit obvious contrast difference in core–shell configuration under SEM observation as shown by the inset. The experimental setup and growth mechanism were detailed in electronic supplementary information (SI, Supplementary Figs. 1–5). Figure 1b shows the low magnification SEM image of as-synthesized ultra-long nanowires with millimeters length on ceramic substrate. These nanowires are found in sufficient bended profile and high quality upon closer view, exposed by the closer scanning electron microscope (SEM) observation in Supplementary Fig. 6. Benefiting from the huge aspect-ratio, they are mechanically uprooted and adjusted to various configurations conveniently. Figure 1c–e corresponds to the SEM images of nanowires aggregations free of impure phases in different shapes after being transferred to Si substrate. Noticeably, except for random pile, these nanowires could align orderly to form oriented bundles with length of ~ millimeter level through simple mechanical treatment. The X-ray diffraction (XRD) analysis upon amounts of nanowires as in Supplementary Fig. 7 implies that the sample is Mn_5_Si_3_ (PDF#42-1285). Low magnification transmission electron microscope (TEM) characterization in Fig. 1f confirms the ultra-long nanowires are consist of single-crystalline core and amorphous shell, as implied by the selected area electron diffraction (SAED) pattern that was composed of sharp diffraction spots and a dispersed circular ring (Fig. 1h). The STEM-EDS mapping analysis in Fig. 1g upon a piece of typical nanowire confirm that the nanowire core is rich of Mn element, while the shell only contains Si and O elements. Figure 1i is the corresponding high resolution TEM (HRTEM) characterization of the nanowire core. A single long nanocable grows in single-crystal along *c*-axis, as examined by combined characterizations with SAED and HRTEM, as in Supplementary Figs. 8–9 and Supplementary Note 2.

### Uniformity and flexibility of Mn_5_Si_3_@SiO_2_ nanocables

**Fig. 2:**
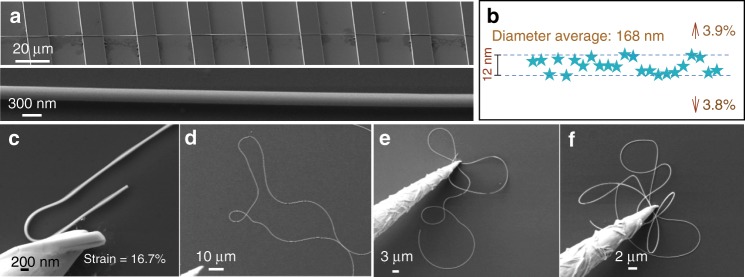
Diameter uniformity and bendability investigation of Mn_5_Si_3_@SiO_2_ nanocables. **a** A ultra-long nanocable on patterned silicon grooves. **b** The thickness distribution along longitude direction. **c** A piece of nanowire bended with 16.7% strain. **d**–**f** The flexibility investigation of a nanocable using a tungsten probe in three-dimensional space.

The dimensional uniformity was investigated upon a typical nanowire that was preplaced on patterned silicon grooves fabricated with lithography process (Fig. 2a), by tracking the total thickness (core and shell) longitudinally. As in Fig. 2b, the thickness evolution displays a maximum fluctuation <4% within a ~300 μm length (data picked evenly spaced). TEM observation implied that the slight fluctuation is attributed to the SiO_2_ shell, while the Mn_5_Si_3_ core displays even better uniformity (Supplementary Fig. 10). Besides features above demonstrated as crystal quality, aspect ratio, uniformity, it is important to deal with considerable deformation without failure as flexible nano-interconect. Normally, high conductive metallic nanowires achieve this in price of irreversible plastic deformation, inevitably resulting in amounts of disorder and subsequent local necking thickness^32^. Electrical degradation occurs accordingly. Alternatively, ceramic Mn_5_Si_3_ is expected to be hard, rigid and brittle at room temperature. With SiO_2_ sheath outside that can disperse huge strain energy^33^, the bendability of Mn_5_Si_3_ is enhanced sufficiently. A tungsten probe was applied to investigate its mechanical performance, as we did previously^34,35^. The picked nanowire displays no fracture even under a bending strain as large as 16.7% (Fig. 2c), which is sufficient enough to be applied as flexible electrical interconnect. A nanowire remains intact even if a three-dimensional complex bending deformation occurred, as in Fig. 2e. A similar bending operation was carried out upon single nanocables on Si_3_N_4_ film with carved microapetures, as demonstrated in Supplementary Note 3, which facilitates the TEM observation. In Supplementary Figs. 11, 12, although the two nanocables experienced bending strain of 11.52% and 13.05%, respectively, there is no fracture behavior in the Mn_5_Si_3_ core. The interesting features, i.e. huge aspect ratio, outstanding uniformity and mechanical flexibility, make Mn_5_Si_3_@SiO_2_ nanocables good candidates for electrical nano-interconnects.

### Fundamental electrical property measurement

**Fig. 3:**
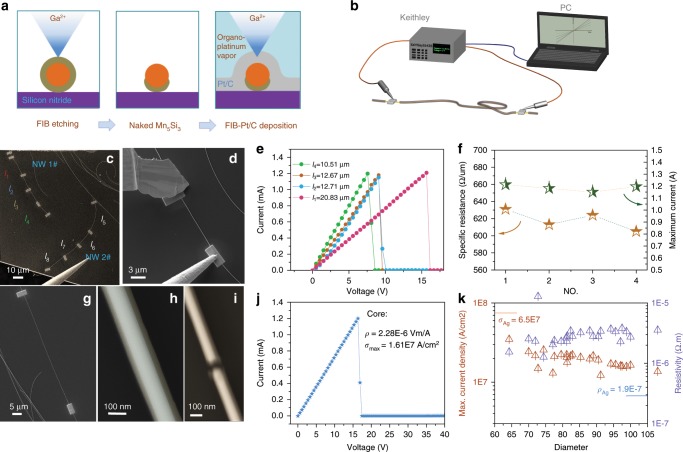
The basic electrical performance investigation of Mn_5_Si_3_@SiO_2_ nanocables. **a** the schematic of double-electrodes device with FIB. **b** The I–V measuring circuit employed in SEM chamber. **c** Two nanocables made into several independent electron channels with various lengths. **d** The testing configuration under SEM observation. **e** Acquired I–V curves of device l_1_–l_4_. **f** The specific resistances and maximum currents corresponding to l_1_–l_4._ **g** A typical device used to determine the resistivity and current density. **h** high magnification SEM image with false color to determine the core thickness. **i** The breaking down point after testing. **j** The I–V curve recorded, which determine a resistivity of 2.28 × 10^−6^ Ω m and maximum current density of 1.61 × 10^7^ A cm^−2^. **k** The values corresponding to 25 nanocables with various thicknesses.

The basic electrical property of the core–shell nanowires were evaluated by fabricating double-electrodes device. The experiment was carried out in crossed dual^−^beams work station (Carl Zeiss Auriga 4523). For device fabrication, a region of the nanowire was firstly exposed to FIB radiation to remove insulative SiO_2_ shell and followed by Pt/C deposition onto resulted naked Mn_5_Si_3_ electrical core (Fig. 3a). Two moveable tungsten probes controlled by independent micro-actuators (Kleindiek MM3A) were in connection with a dual^−^channel sourcemeter (Keithley 2634B) to acquire I–V characteristics (Fig. 3b). Several devices with different channel lengths were made in single nanowires to examine the uniformity of electrical performance across the total nanowires longitudinally (Fig. 3c). Figure 3d shows the measuring configuration corresponding to l_3_ device, where one of the tungsten probes has been machined by FIB. During the test, a sweeping voltage was loaded until the nanocable failed. The linear I–V characteristic in Fig. 3e demonstrates a good ohmic contact between the electrical core and the Pt/C electrode, according to which the resistivity and maximum current density of Mn_5_Si_3_ core in l_1_–l_4_ were determined, as shown in Fig. 3f. Another nanowire including three devices is also analyzed as in Supplementary Fig. 13. It is found that in total length both of the nanowires delivers homogeneous electrical performance, i.e. maximum currents tolerant and specific resistance. It is accordingly sufficient to investigate the electrical property in statistical manner of many nanowires with various diameters, for which the figure or merits could be evaluated. For example, as in Fig. 3j–k, a nanowire with 97 ± 3 nm core was made into a 58.5 μm length channel device. The resistivity was determined to be 2.28 × 10^−6^ Ω m and maximum current density of 1.61 × 10^7^ A cm^−2^, respectively. Totally 30 nanowires with various thicknesses (62–102 nm) were investigated in the similar way, as presented in Fig. 3k. For all the nanocables examined, they output electrical performance with resistivity of 1.28–3.84 × 10^−6^ Ω m, and maximum current density of 1.22–3.54 × 10^7^ A cm^−2^, respectively. The resistivity is at the same level to bulk Mn_5_Si_3_ and crystal films reported by other researchers^27,36,37^. It is worthy to be mentioned that the values for thinner nanowires are more fluctuated, which may originate from the more significant percentage error during dimension determination. For comparison, the electrical figure of merits of Ag nanowires was investigated in the same way, which was marked in Fig. 3k. The mean values are 1.7 × 10^−7^ Ω m and 6.5 × 10^7^ A cm^−2^, respectively.

The stability of its electrical property under dynamic deformed condition was investigated by employing an in situ I–V measurement of a single nanocable, as in Supplementary Fig. 14. One end of the nanocable was fixed onto a tungsten probe using electron beam assisted Pt/C deposition after removing the SiO_2_ shell (Supplementary Note 4). The other end was connected to Au pattern in the same way. Five I–V curves (corresponding to different bending states) in total were acquired, as in Supplementary Fig. 14h. It is interesting that the electrical property appears robust, although the nanocable was in complex bending states.

### The electrical performance at high temperature

Except for the electrical performance related to electrons transport, robust stability resistant to thermal, structural, and oxidative damage are of significant importance in nanoscale electrical interconnects, where it is much more serious than their bulk counterparts due to significantly larger percentage of surface atoms with higher reactivity^38,39^. Although with prominent conductivity and highly developed fabrication technology, Ag and Cu NWs are deficient to apply in high temperature, acidic, and oxidative environments as a result of their poor stability^40^. With uniform protective sheath, the core–shell Mn_5_Si_3_@SiO_2_ nanocables may display both the electrical performance of Mn_5_Si_3_ core and physicochemical stability of the SiO_2_ shell. Below, we systematically address these items combining with rational experimental design.

**Fig. 4:**
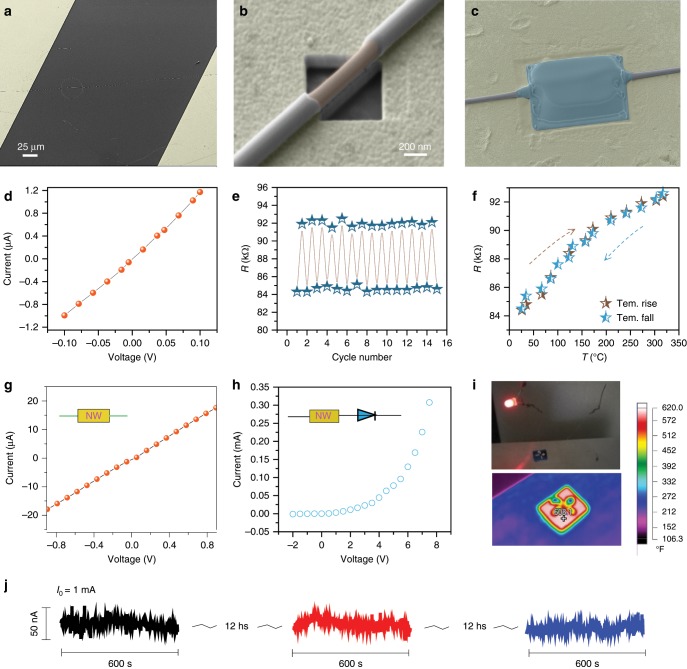
Resistance investigation at evaluated temperatures of Mn_5_Si_3_@SiO_2_ nanocables. **a** The real device with 250 um channel length for experiment. **b** The electrode region after FIB radiation with Mn_5_Si_3_ core naked. **c** After Pt/C deposition and connection to Au patterns. **d** The I–V curve acquired. **e** The recorded resistances as the temperature switching between 317 °C and 26 °C. **f** The resistance-temperature dependence at a range of 26-317 °C. **g** The I–V curve of another device used for long-time stability investigation at ~300 °C. **h** The I–V curve of the device incorporating a red LED light. The insets show the illustration of the nanowire connection. **i** The optical picture of the circuit (upper) and thermal imaging of the device substrate (bottom). **j** the long-time stability investigation at 300 °C.

Electrical performance at raised temperature in the air was firstly investigated, which is to evaluate its ability to resist high temperature and air oxidation. A set of experiments were schemed rationally and carried out, as described in Supplementary Note 5. Figure 4a shows the as-fabricated device with channel length of ~250 μm and nanowire dimension of 150 nm (core) and 206 nm (shell) respectively (magnified device image shown as Supplementary Fig. 15). The electrode contacts were made sequentially by SiO_2_ sheath removing and Pt/C deposition as shown in Fig. 4b–c. The typical I–V characteristic acquired at room temperature as in Fig. 4d implies the resistance is about 84 kΩ. Nextly, a hot plate was used to evaluate the substrate temperature. The value of upper surface of the substrate was identified to correspond to the exact temperatures of the nanowire, which were calibrated by a thermal imager (Fluke TiS55), as in Supplementary Fig 16. The resistances at various temperatures were then determined by an electrical multimeter. As in Fig. 4f, the resistance values increased for 14.45% (from 84.42 to 92.62 kΩ) as the temperature raised to 317 °C from 26 °C. Noticeably, the R-T dependences obtained from measurements under heating and subsequent cooling condition exhibits good coincidence within a temperature change loop (as marked by dash arrows). It indicates that the Mn_5_Si_3_ core deliver a metal^−^like conductive characteristic, although it has a very narrow band gap. Moreover, the resistance responded rapidly as the temperature switched between 26 °C and 317 °C (Fig. 4e). Such an R-T performance implies that Mn_5_Si_3_@SiO_2_ nanocables not only behave robust as high temperature nano-interconnect in the air, but is of potential as a wide-range thermometer working in atmospheric environment. Noticeably, 317 °C is not the ultimate temperature tolerable of the nanowire, which is close to the maximum value that the Au/Ti electrodes can withstand (Supplementary Fig. 17). In order to determine the maximum temperature they can bear, many nanocables were annealed in air condition for 2 h. The morphorlogy of them appeared unchanged after annealing treatment at 500 °C as shown in Supplementary Fig. 18a, b. I–V measurement upon one of a random nanocable indicated that it was still electrical available (Supplementary Fig. 19). When the annealing temperature increased to 600 °C, the Mn_5_Si_3_ core began to degenerate (Supplementary Fig. 18c, d). As reference, the thermal resistance of Ag NWs in the air was investigated as in Supplementary Fig 19, which implies that many of the nanowires are broken when temperature is higher than 160 °C.

A long-time stability of a single nanocable device (channel length of ~75 μm and core diameter of ~81 nm) at high temperature was examined. Figure 4g shows the I–V characteristic of the device and Fig. 4h corresponds to the total circuit with a red LED light incorporated. The current of the circuit was ~1.1 mA at room temperature when driven by 8.5 V voltage, which was rapidly heated by a hot plate to ~300 °C (upper surface) checked by a thermal imager (Fig. 4j). Figure 4i shows the optical image of the working circuit including LED light, nanowire component and other electrical connecting wires. A current evolution for ~24 h at targeted temperature was investigated as shown in Fig. 4i–j, where three I–t evolution (*I*_0_ = 1 mA) curves were recorded with equivalent time interval. Interestingly, the electrical performance displayed excellent stability with a very slight current fluctuation, and no degradation was observed even comparing to the room-temperature examination.

### Electrical availability in acidic and oxidic environment

**Fig. 5:**
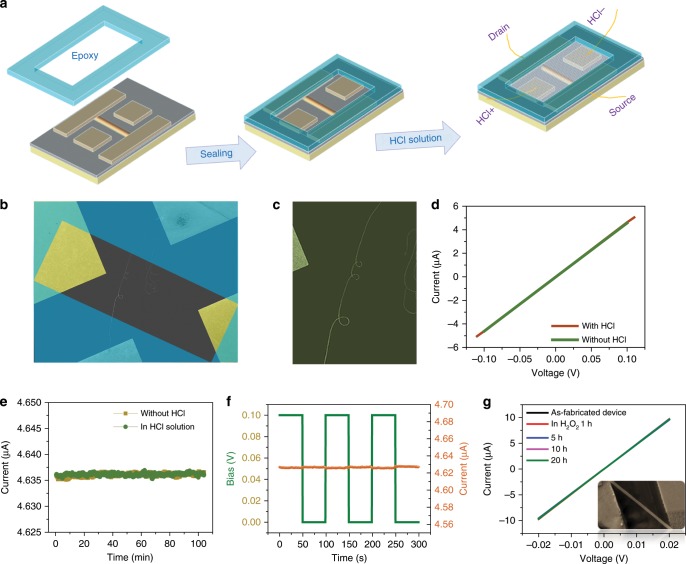
Electrical availability to HCl and H_2_O_2_ environments. **a** The fabrication schematic of the device for HCl shielding test. **b** Real device fabricated, the regions in aqua green would be sealed with epoxy. **c** The closer view of naked nanocable. **d** The I–V curves of the device with/without HCl solution filling. **e** The current-time evolution at constant voltage with/without HCl solution filling. **f** The current evolution (brown) driven by a constant voltage with a periodic pulsed electrical voltage (green) applied between HCl^+^ and HCl^−^.**g** The I–V curves of the nanocable (inset) after being immersed in H_2_O_2_ solution for 1, 5, 10, and 20 h, respectively. Closer observation of the nanocables is shown in Supplementary Fig. 20b.

Device working under humid environment is not only faced with risk to be oxidized, but corroded by acidic species. The Mn_5_Si_3_@SiO_2_ nanocables are expected to possess outstanding resistance by integrating the chemical characteristic of SiO_2_. The performance of acidic corrosion resistance was investigated upon a nanowire device (channel length of ~1 mm) sealed with epoxy, leaving only the channel naked, as illustrated in Fig. 5a. A double electrode (HCl + and HCl^−^) were fabricated at the nanowire sides to load electrical field to the solution. Accordingly, when acid solution (HCl PH = 1) filling the groove, only the nanocable and HCl electrodes was exposed to acidic condition, while the nanowires electrode contacts was insulated. Figure 5b is the real nanowire device before sealed and Fig. 5c is the closer view of the nanowire channel. The electrodes (Pt/C-Au contacts) configurations of the device were shown in detail as Supplementary Fig. 20a. The I–V curves in Fig. 5d were acquired with/without HCl solution respectively, which displayed almost the same profile. The I–t evolution of the nanowire with/without HCl immersion was further monitored with a constant 0.1 V voltage respectively, as in Fig. 4e. Noticeably, the currents in both cases display a stable output, which implies that the conductive nanocable works free of disturbance from HCl solution, i.e. possible chemical corrosion and current leakage. An extreme surrounding condition with co-existence of complex electric field, ionic current and strong acidity was simulated by loading a periodic rectangular pulse field (peak voltage of 0.1 V) between the HCl^+^ and HCl^−^. As in Fig. 5f, the current evolution behave stable with very slight fluctuation. These tests demonstrate the core–shell nanocables are suitable to work under strong acidic, complex field and current conditions. Oxidation resistance experiment was further carried out.

Weak oxidation resistance is the most serious problem for metallic electrical wire. Yu’s group has reported that Cu NWs can be quickly oxidized by dissolved oxygen within several hours, which degenerate and evolve into a mace-like structure when stored in water or nonpolar organic solvents^17^. Here we employed a parallel test of Ag NWs and synthesized Mn_5_Si_3_@SiO_2_ nanocables upon the chemical stability in highly oxidizing H_2_O_2_ solution (10%). After 2 h soak in a liquid drop, Ag NWs suffered from serious damage as in Supplementary Fig. 21, while the Mn_5_Si_3_@SiO_2_ nanocables remained intact even experienced a 20 h treatment (Supplementary Fig. 22). To examine the electrical availability, a device composed of two shoulder-by-shoulder nanowires were soaked in in H_2_O_2_ solution (10%). During a 20 h time, the device was got out to execute I–V measurement at several point-in-times, as in Fig. 5g. The electrical performance always remains robust without any degeneration all over the experiment.

In fields like chemical sensors, bioelectronics, MEMS and also many other functional devices, both the electrical nanowires and working material are specifically exposed to the complex occasions, i.e. ionic solution, acidic, high temperature, high power, bendable, and stretchable functional systems. The SiO_2_ sheath sufficiently shields potential structural and function damage to the electrical core. Moreover, with good biological compatibility, a single long nanocable is possible to execute intercellular and intracellular task by being made into a probe system in micro and sub-micro scale. Also, as telescopic electrical component in NEMS/MEMS system, it can work free in three-dimensional space without constraint from substrate. The electrical contact could be fabricated via common silicon based micro/nanotechnologies developed.

## Discussion

In summary, a CVD growth in molten glassy matrix of Mn_5_Si_3_@SiO_2_ nanocables with high quality, ultra-large aspect ratio and outstanding uniformity was developed, which integrate the electrical property of Mn_5_Si_3_ core and also the chemical/physical stability of SiO_2_ shell. Benefiting from the complete and uniform wrapping of inert SiO_2_ sheath, the nanocables exhibit amazing electrical performance in extreme environment, including high temperature (higher than 317 °C), complex electric field (current) and acidic condition. Comparing to common nanoscale electrical interconnects candidates (i.e. Ag and Cu), Mn_5_Si_3_@SiO_2_ nanocables undoubtedly display more promising applied in future integrated electronic device, bioelectronics and special device working in harsh environments. Noticeably, single nanowires are capable of dealing with seriously bending deformation, which withstood a strain of 16.7% free of failure. Due to the rigid characteristic of ceramic Mn_5_Si_3_, no plastic event would occur in electrical core, which means the improved bendability free of electrical depreciation. This feature is important for application in flexible device and in complex wiring condition. The Mn_5_Si_3_@SiO_2_ nanocables are compatible to silicon based semiconductor technologies. Highly developed micro-nanomachining technologies like reactive ion beam etching, UV lithography and electron beam lithography can be applied to remove the insulating SiO_2_ shell in specific region and fabricate electrode contacts.

## Methods

### Sample fabrication

Experiment A was firstly carried out to create Mn-Si-O rich region. Mn and SiO powder with molar ratio of 6:1 was grinded in a mortar, until they were mixed homogeneously, which was applied as the sole reacting precursor (900 mg). The mixture on a ceramic sheet was placed in a protecting tube. The whole assembly was then pushed into the furnace with the powder at the center of the heating region. After a forvacuum process, the furnace was heated to 1250 °C in 60 minutes, which was held for 3 hours. H_2_ at flow rate of 100 sccm was introduced to the chamber as soon as the heating procedure began until the pressure reached 20 kPa. The gas in the chamber was exhausted by a mechanic pump after the reaction, meanwhile the furnace cooled to room-temperature with help of water coolant. Experiment B was then employed to synthesize the sample. 90 mg mixture of Mn (99% purity, Aladdin) and Si (99.9% purity, Aladdin) powders in molar ration of 1:3 and 100 mg SiO (99.99% purity, Aladdin) were used as precursors. Both of them were placed on a new ceramic sheet with the Mn-Si powder at the center of the high temperature region. A similar heating procedure and atmosphere condition were applied during the reaction.

### Device fabrication and measurement

These operations were carried out in crossed dual-beam work station (Carl zeiss Auriga 4523). Two tungsten probes linked to micro-actuators with minimum step superior to 100 nm in three dimensions were applied to manipulate a single nanowire. They are also connected to external circuit, which could transport electrical signal with a current noise at the level of ~pA. To remove the SiO_2_ sheath of the nanocaple and meanwhile ensure the electrical core intact, a focused Ga^2+^ beam with a current density of ~5 pA μm^−2^ was used to machining for 10 s. FIB (~pA μm^−2^, 120 s) induced chemical vapor deposition of Pt-C block was then executed on the exact surface through driving organic platinum gas to decompose with help of GIS (Gas Injection System). The I–V characteristics could be acquired as soon as the two tungsten probes move to contact the Pt/C block. During the test, electron beam radiation should be blank to avoid unwanted electron injection effect. The channels in Figs. 4 and 5 were fabricated using a lithography and lift-off technology. Au (90 nm)/Ti (10 nm) was applied as electrodes pattern for external circuit connection.

## Supplementary information

Supplementary Information

## Data Availability

The authors declare that the data supporting the findings of this study are available from the corresponding author upon reasonable request.
