# Supplementary material for: Millimeters long super flexible Mn5Si3@SiO2 electrical nanocables applicable in harsh environments
Source: Nat Commun. 2020 Jan 31;11:647. doi: 10.1038/s41467-019-14244-5 (PMC6994472; doi:10.1038/s41467-019-14244-5)
Supplement: Supplementary file 1 — Supplementary Information [file 41467_2019_14244_MOESM1_ESM.pdf]

Supplementary information for

Millimeters Long Super Flexible $Mn_5Si_3@SiO_2$ Electrical Nanocables Applicable in Harsh Environments

*Yong Sun, Bo Sun, Jingbo He, Guowei Yang & Chengxin Wang**

State Key Laboratory of Optoelectronic Materials and Technologies, School of Materials Science and Engineering, Sun Yat-sen (Zhongshan) University, Guangzhou 510275, People's Republic of China

E-mail: wchengx@mail.sysu.edu.cn

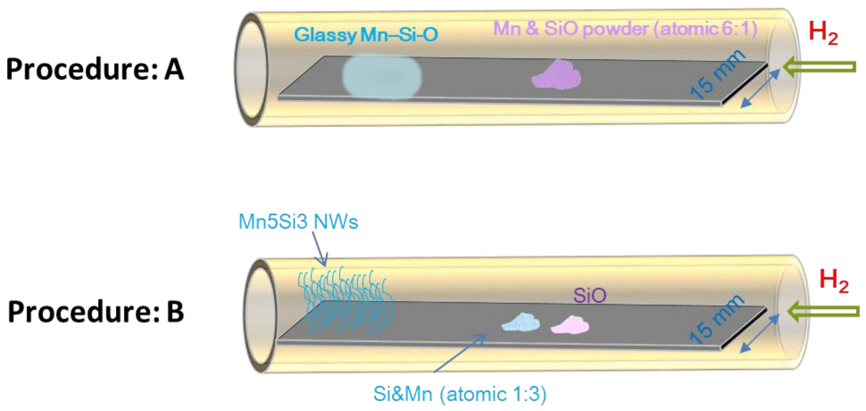

Supplementary Figure 1 | Schematic of the growth (experiment A and experiment B) of Mn_5Si_3@SiO_2 nanocables.

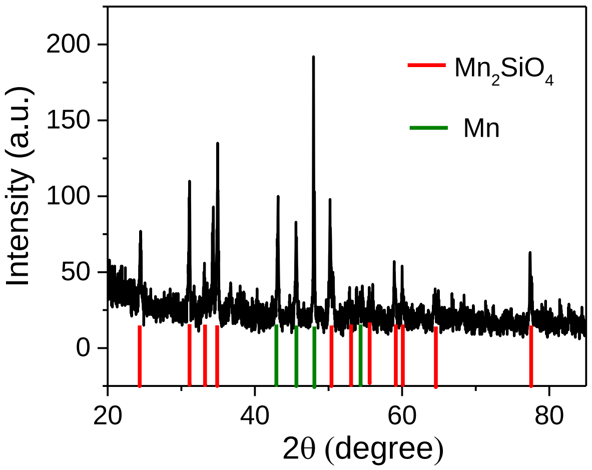

Supplementary Figure 2 | The XRD pattern of Mn&SiO mixture after thermal reaction for 60 minutes at 1100℃.

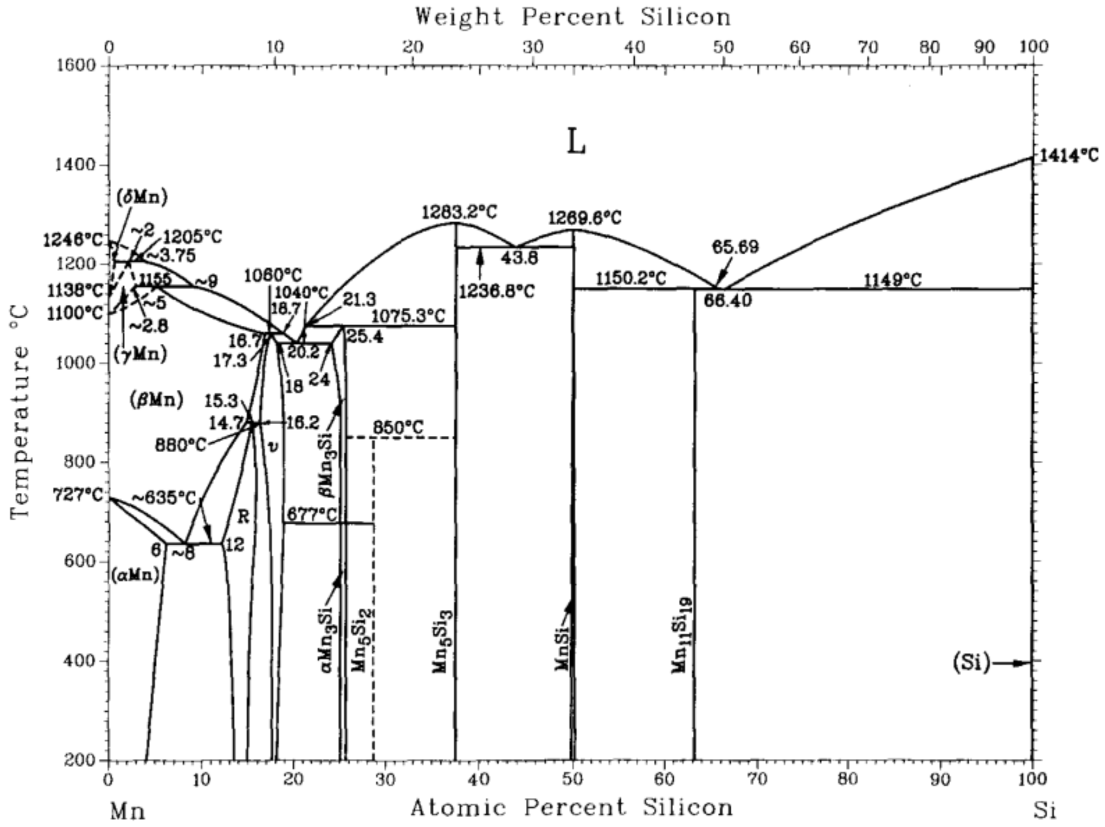

Supplementary Figure 3 | Binary phase diagram of Mn-Si system. Copyright © 1991, ASM International.

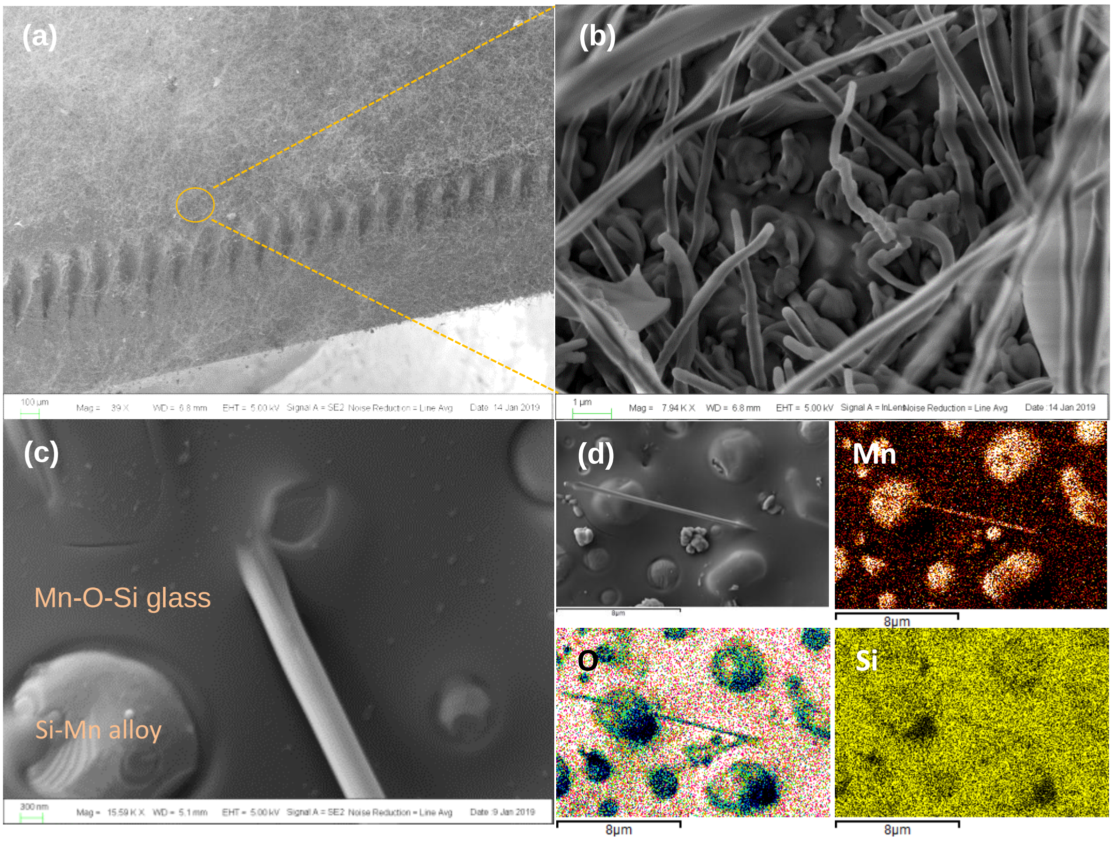

Supplementary Figure 4 | SEM and EDS analysis of the growth process. (**a**) SEM image in very low magnification of an as-synthesized sample (nanocables on ceramic substrate). (**b**) The closer view of marked region. (**c**) SEM observation of a nanowire grown from the Si-Mn-O matrix. (**d**) Elemental distribution of Mn, O and Si acquired by EDS mapping.

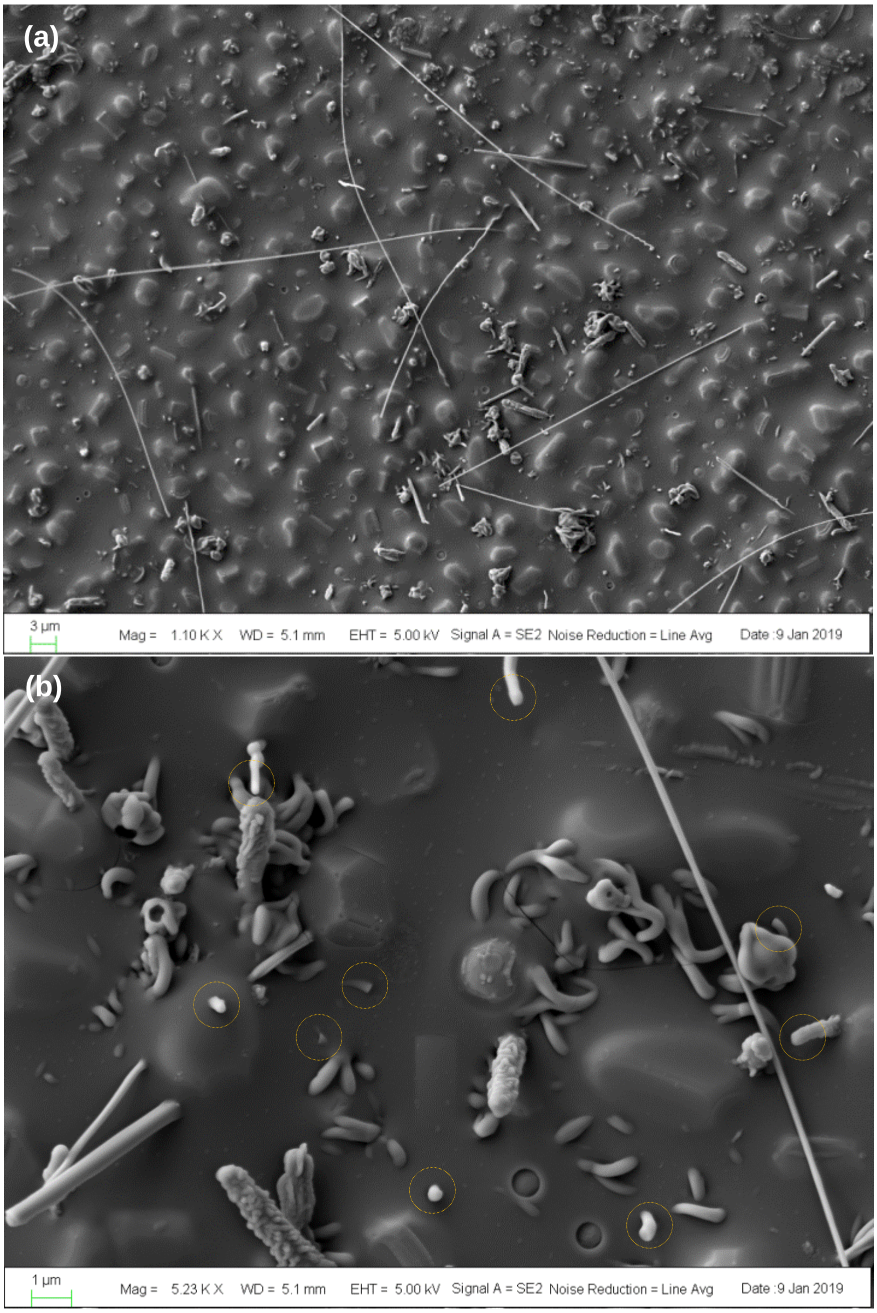

Supplementary Figure 5 | SEM characterization of fractured surface of the sample. (**a**) The surface morphology characterization of the sample after most nanocables being uprooted mechanically. (**b**) The highlighted fracture surface of some nanocables.

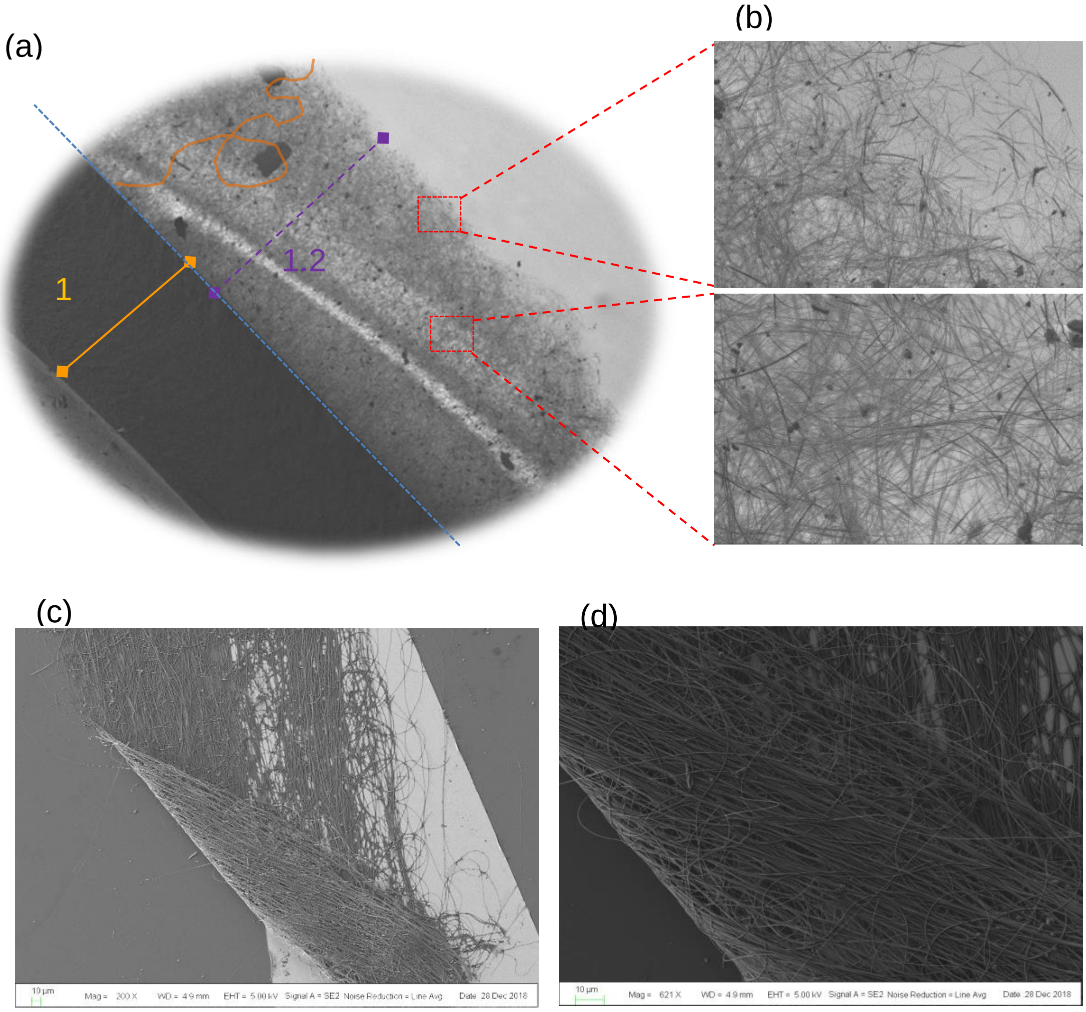

Supplementary Figure 6 | SEM characterization of as-synthesized interlaced nanowires. (**a**) the cross-section SEM observation of as-prepared sample. (**b**) SEM images corresponding to marked region in (a). (**c-d**) nanowires film composed of thousands of oriented nanowires, which was made with simple mechanical manipulation.

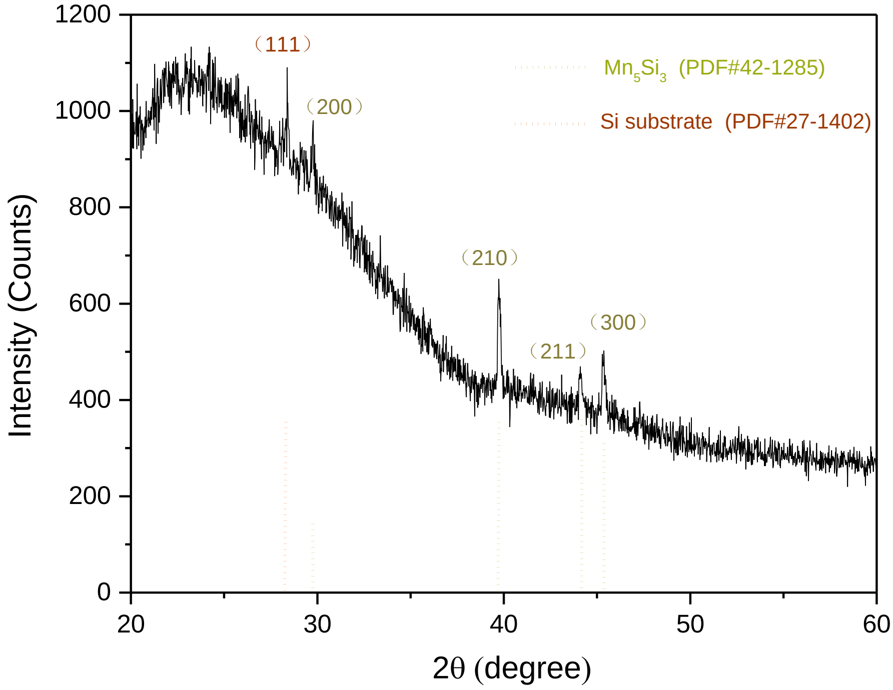

Supplementary Figure 7 | XRD characterization of the sample.

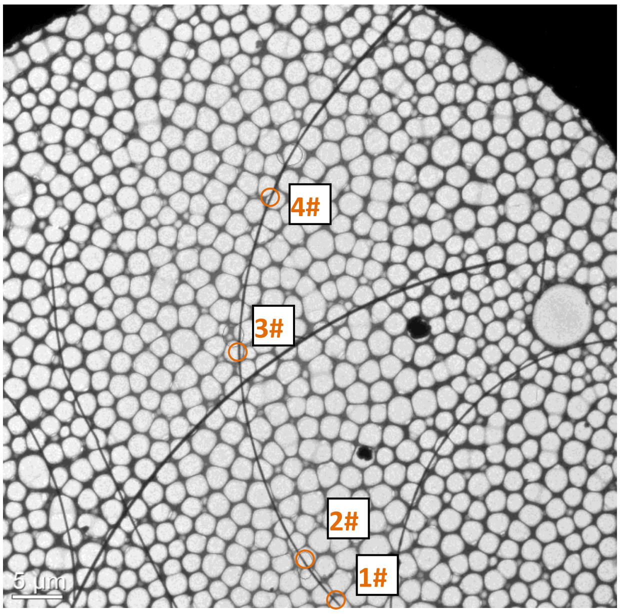

Supplementary Figure8 | Low magnification TEM image of the nanocable used for SAED and HRTEM characterization.

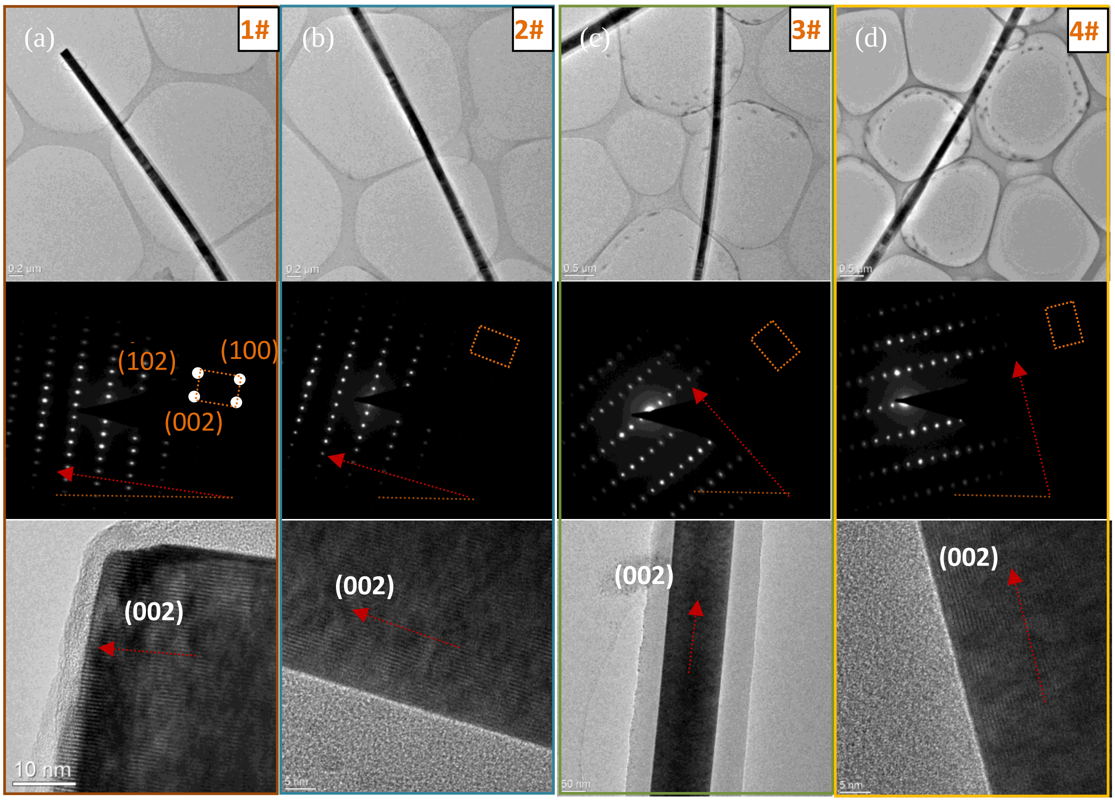

Supplementary Figure 9 | TEM characterization of a long nanocable. (**a-d**) Low magnification images, SAED patterns and HRTEM images corresponding to region 1#, 2#, 3#, and 4# respectively, as marked in Supplementary Figure 8.

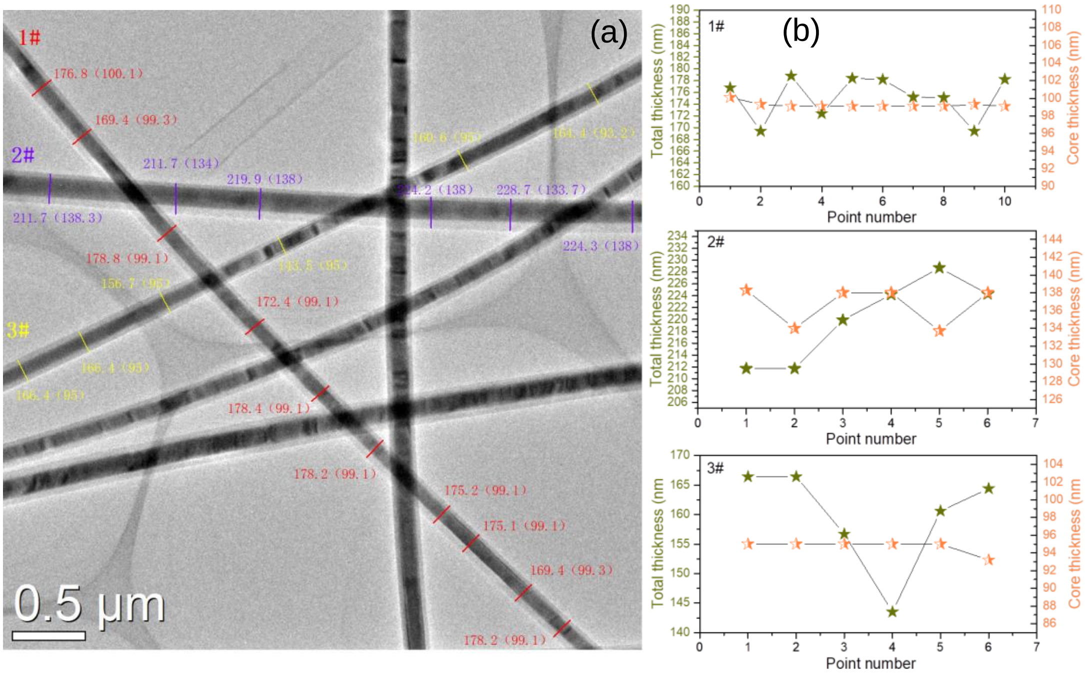

Supplementary Figure 10 | Dimension uniformity investigation of these nanocables. (**a**) TEM image of several typical nanocables. (**b**) The thicknesses (core and total diameters) distribution of three components.

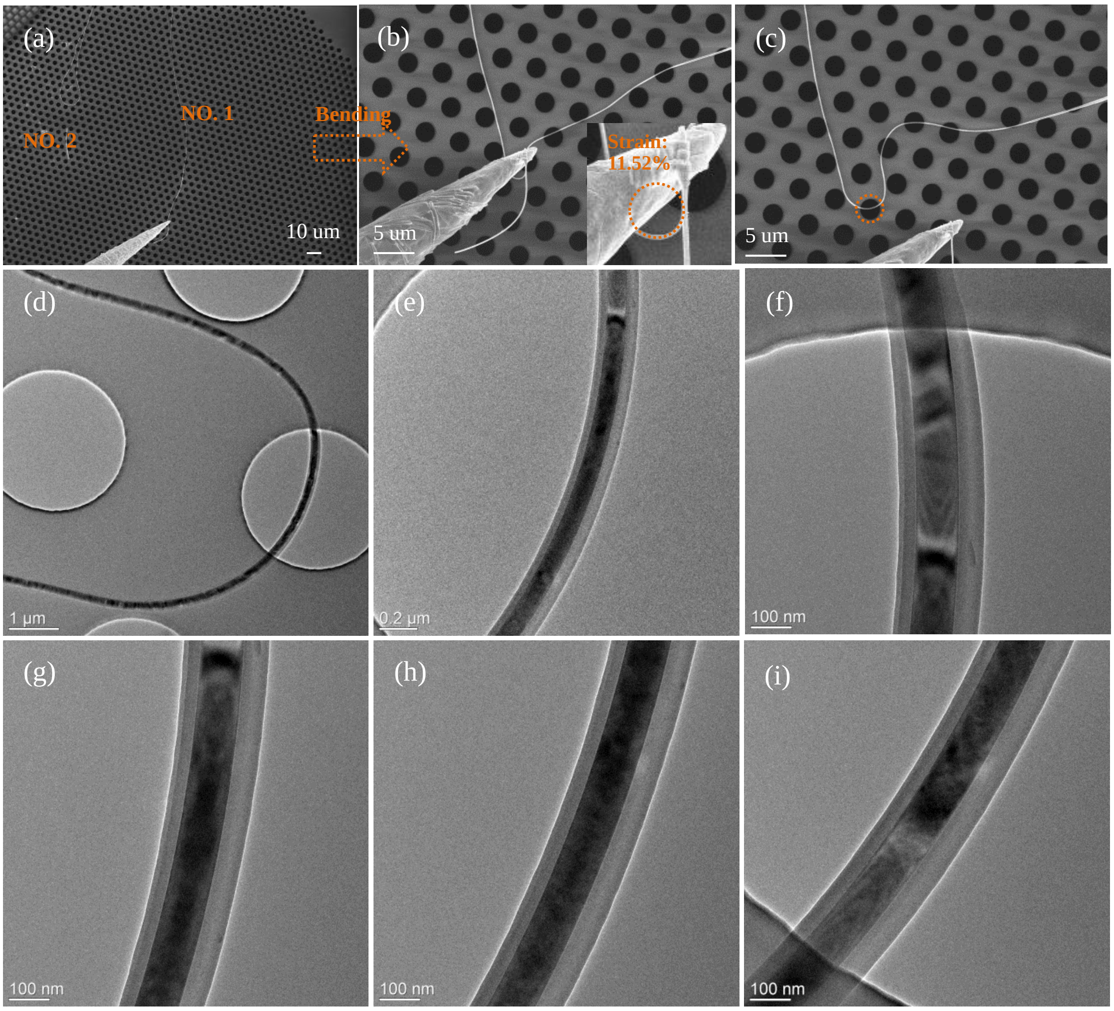

Supplementary Figure11 | Combined SEM and TEM analysis of a bended nanocable. (**a**) SEM image of the two nanocables applied bending experiment. (**b**) The NO. 1 nanocable bended with strain of 11.52%. (**c**) SEM image after the tungsten probe retracted. (**d**) The low magnification TEM image of the nanocable as in (**c**). (**e-i**) TEM images of segments experienced maximum bended strain as marked in (c).

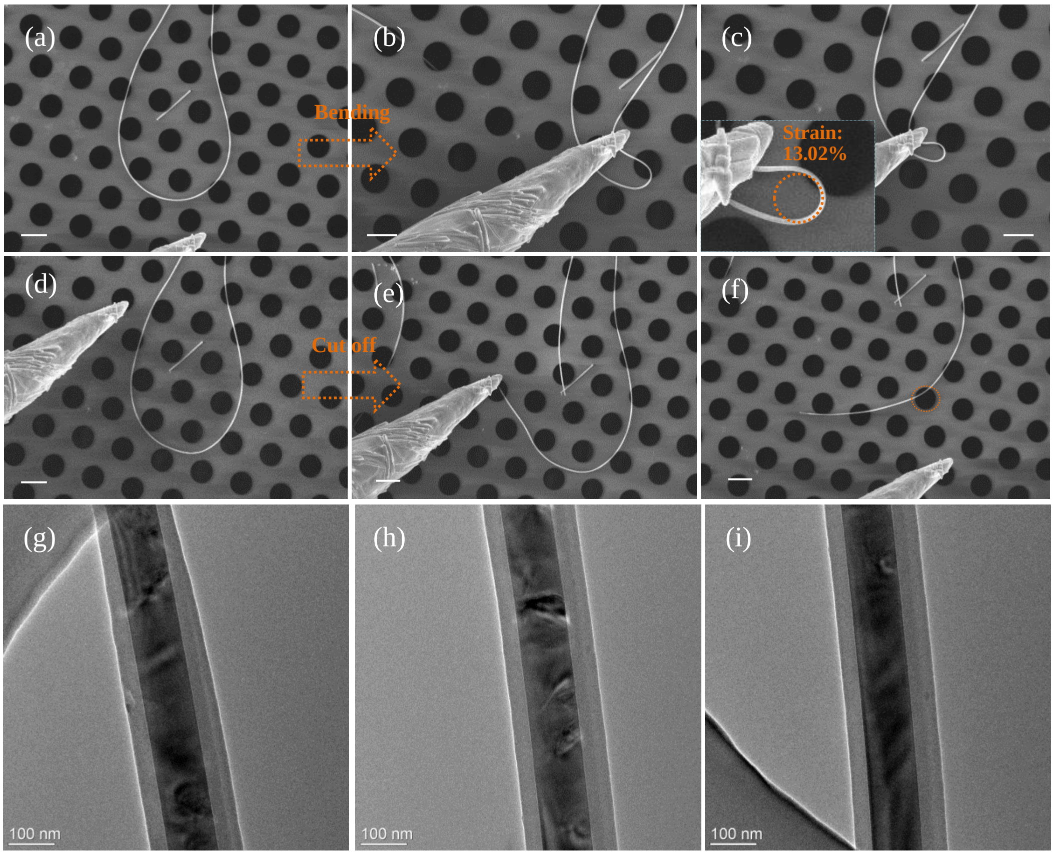

Supplementary Figure12 | Combined SEM and TEM analysis of another bended nanocable. (**a**) SEM image of the No. 2 nanocable before bending. (**b-c**) Two bending configurations. (**d**) After the probe retracted. (**e-f**) The nanocable was cut off and moved the maximally strained segments to carved region of the Si_3N_4 film.

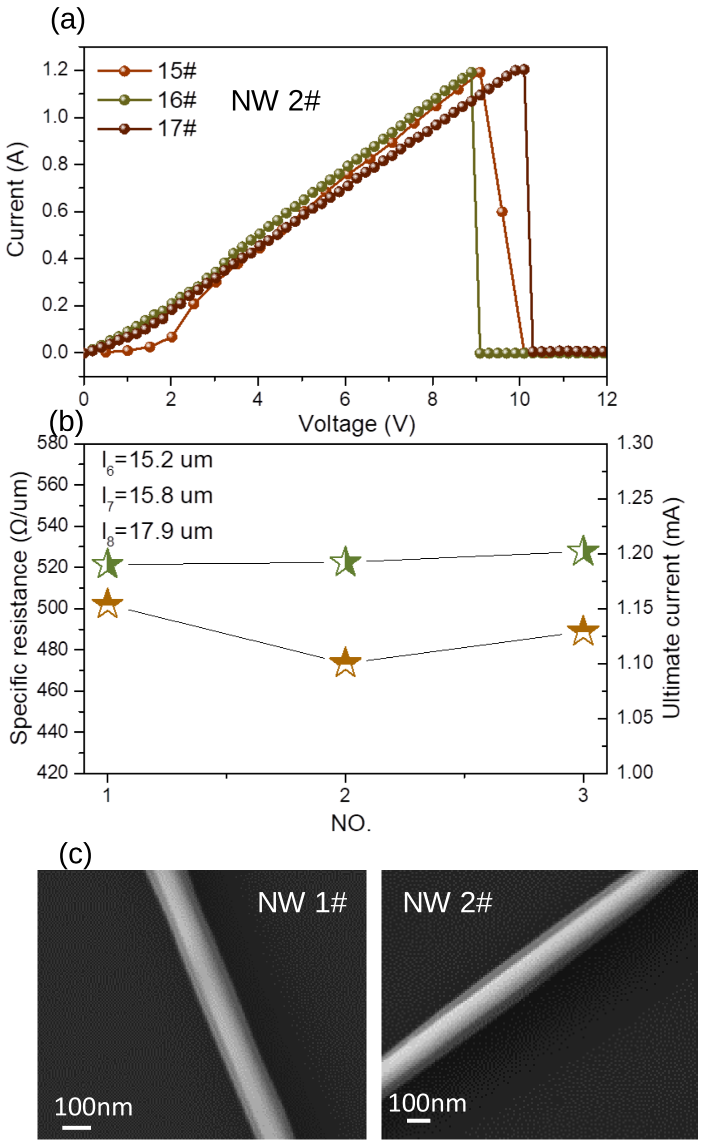

Supplementary Figure 13 | Electrical performance of NW 2#. (**a**) I-V curves of three devices made from NW 2#. (**b**) Specific resistance and maximum current. (**c**) SEM images of the two nanowires used.

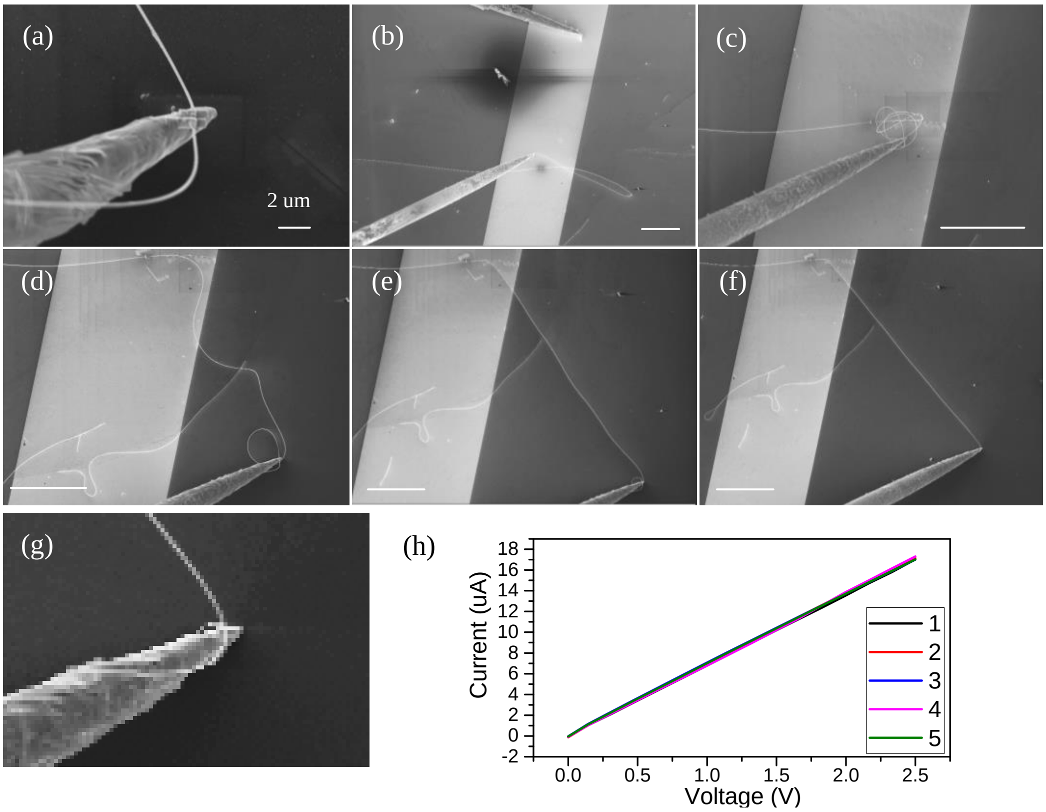

Supplementary Figure 14 | **In situ I-V measurement of a nanocable under bending state**. (**a**) The nanocable-probe contact fabricated using electron beam assisted Pt/C deposition. (**b**) One probe can freely to fold the nanocable and the other probe contact the Au pattern for I-V acquirement. (**c**) The nanocable was bended into complex configuration. (**d-f**) The nanocable was stretched until it was almost straight. (**g**) Magnified image of the probe tip position: a micro circle (~2 um) wrap the tip closely. (**h**) Acquired I-V curves of the nanocable corresponding to (b-f).Scale bar 50 um.

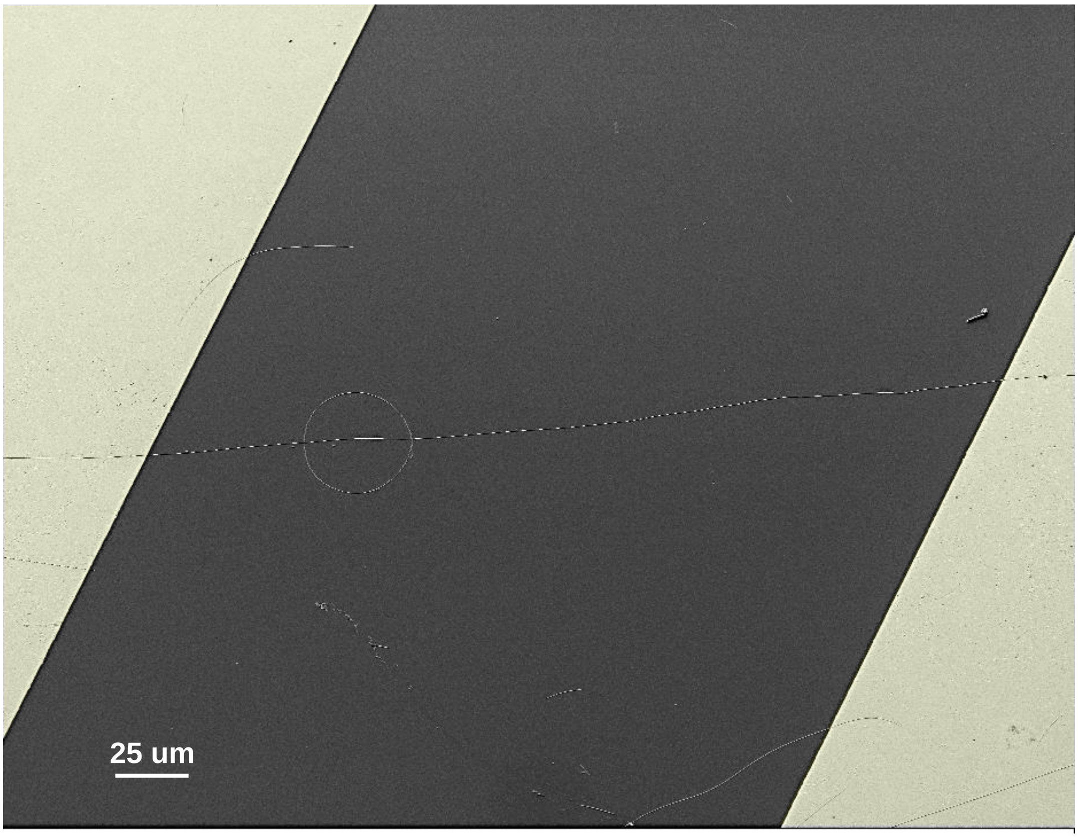

Supplementary Figure 15 | Magnified device SEM image corresponding to Fig.4a.

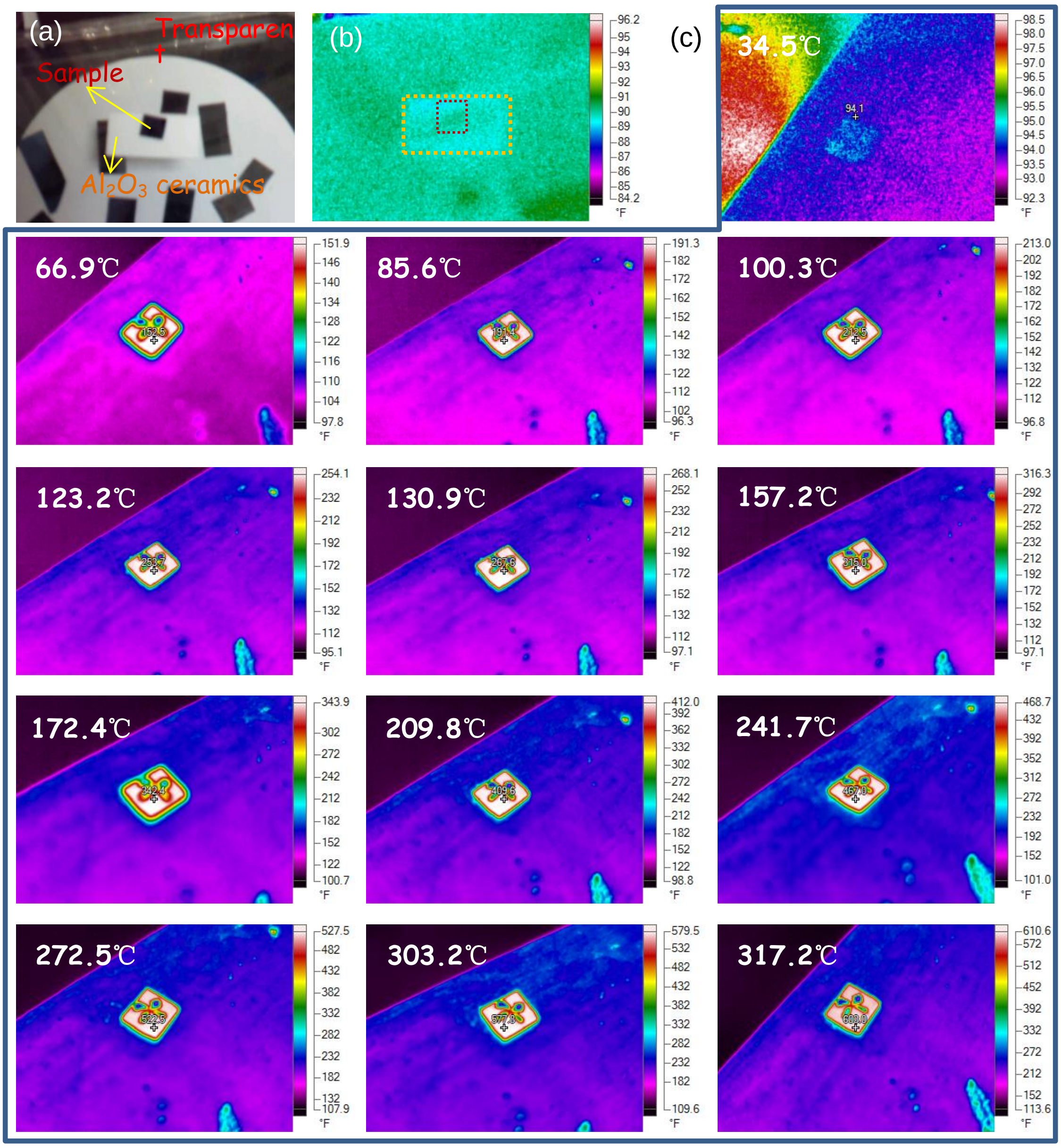

Supplementary Figure 16 | Temperature measurement of device surface. (**a-b**) The emissivity calibration of the substrate used, in which ceramic sheet and plastic as references. (**c**) The thermal imaging of the substrate at various temperatures corresponding to Figure | 4f.

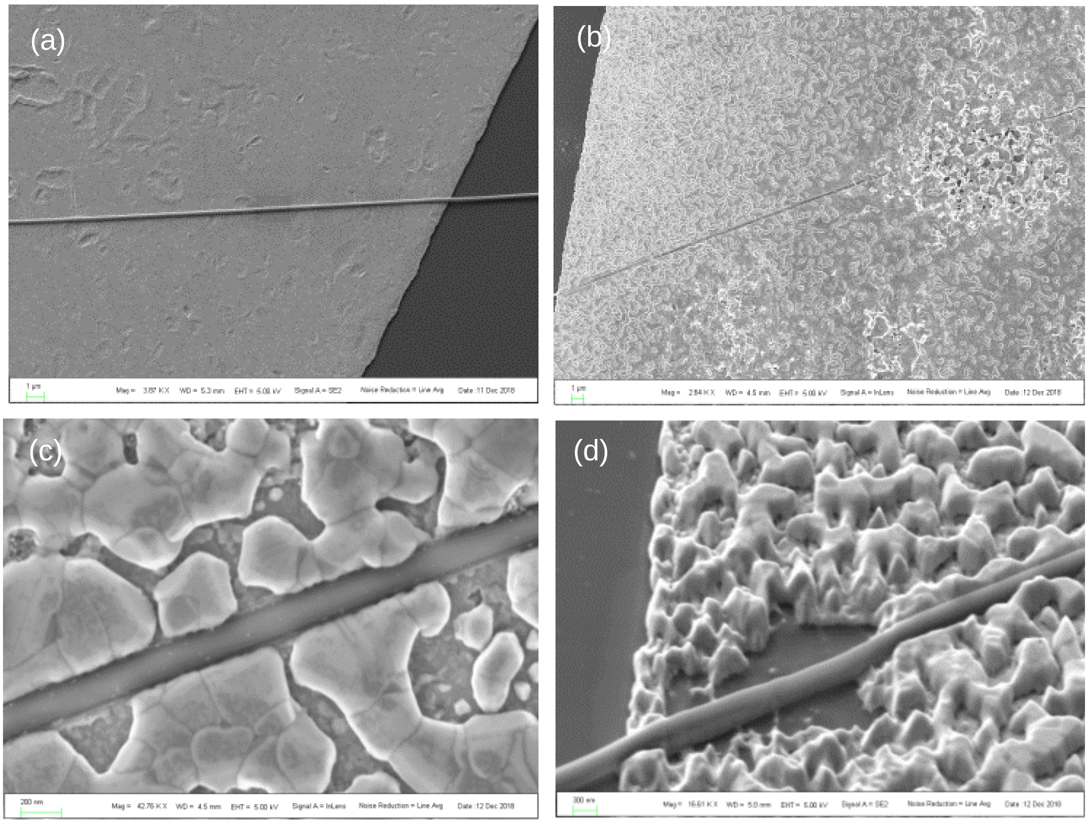

Supplementary Figure 17 | The change of Au electrodes at elevated temperatures. (**a**) SEM image of original Au pattern before high-temperature test. (**b**) Surface morphology of Au after 317℃ treatment. (**c**) SEM observation in higher magnification. (d) SEM image recorded after the platform tilted for 54 °.

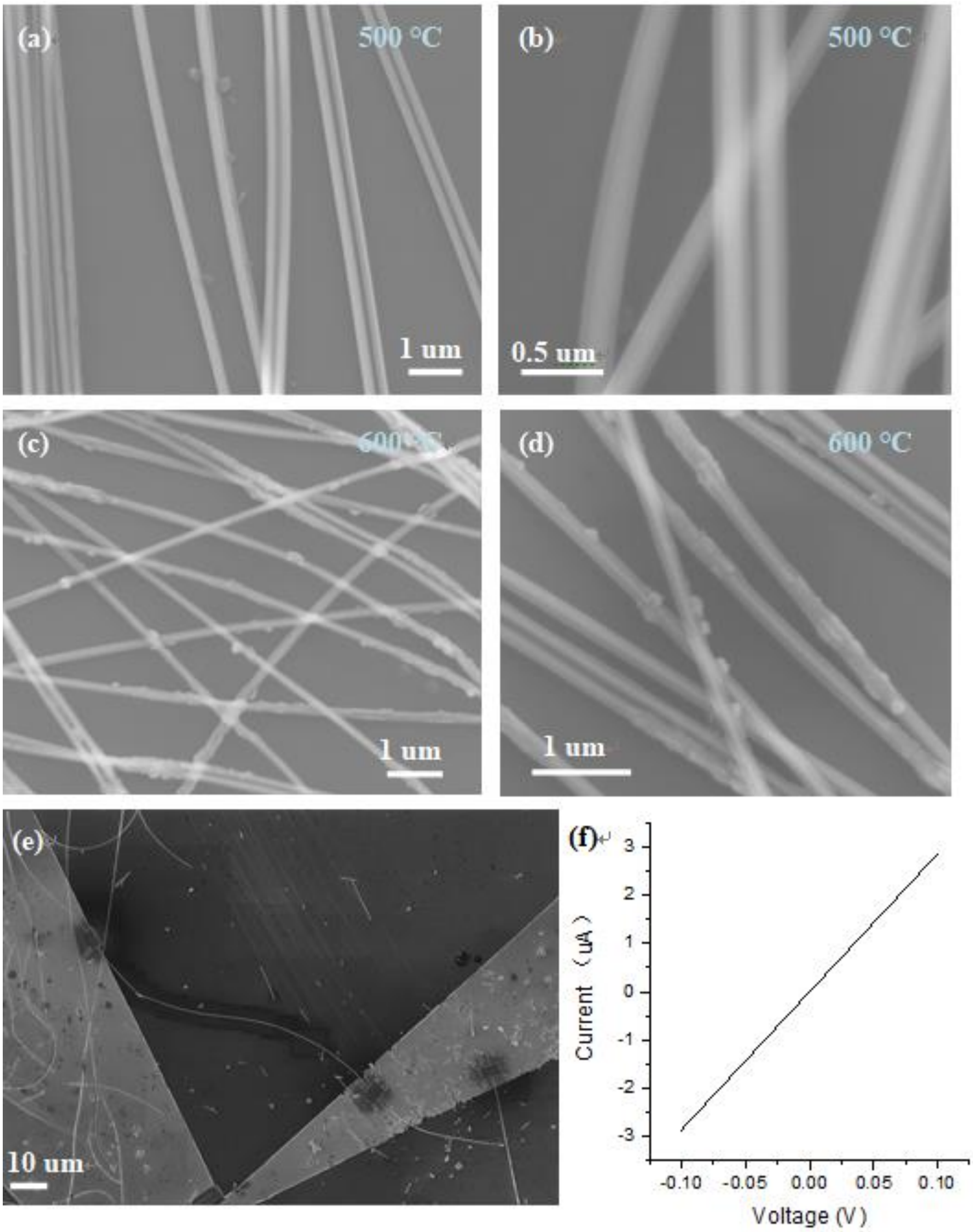

Supplementary Figure 18 | SEM images of $Mn_5Si_3@SiO_2$ nanocable after annealing peocess. (**a-b**) SEM images of the nanocables after 500 ℃ 2 hours in air atmosphere. (**c-d**) SEM images of the nanocables after 600 ℃ 2 hours in air atmosphere. (**e-f**) Electrical examination of the nanocable after annealed at 500 ℃.

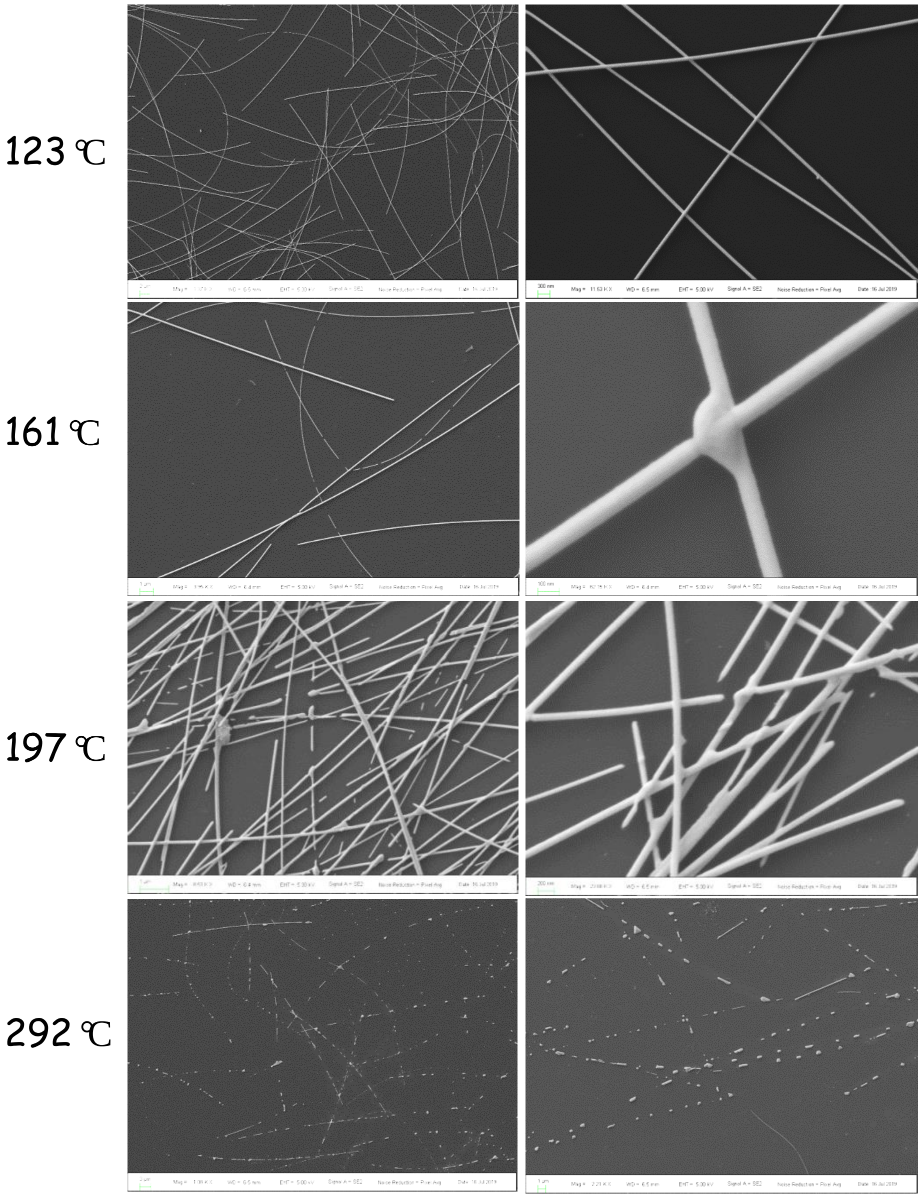

Supplementary Figure 19 | SEM images of Ag NWs after thermal treatment in the air at various temperatures for 20 minutes.

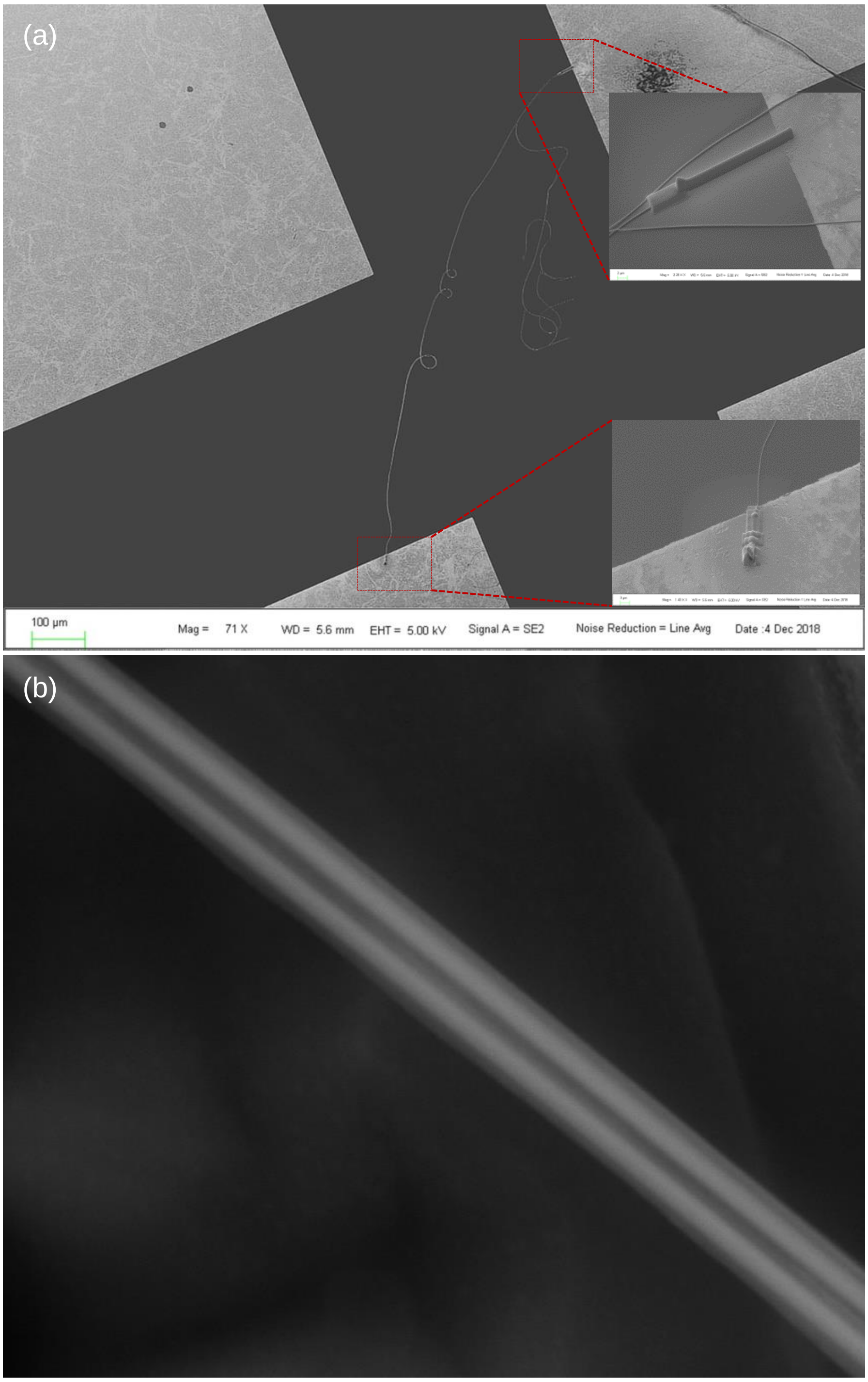

Supplementary Figure 20 | SEM images of device used. (**a**) Original SEM image of device applied for HCl resistance test, the insets shows the electrodes configuration. (**b**) SEM image in high magnification of the nanowire device used for H_2O_2 treatment.

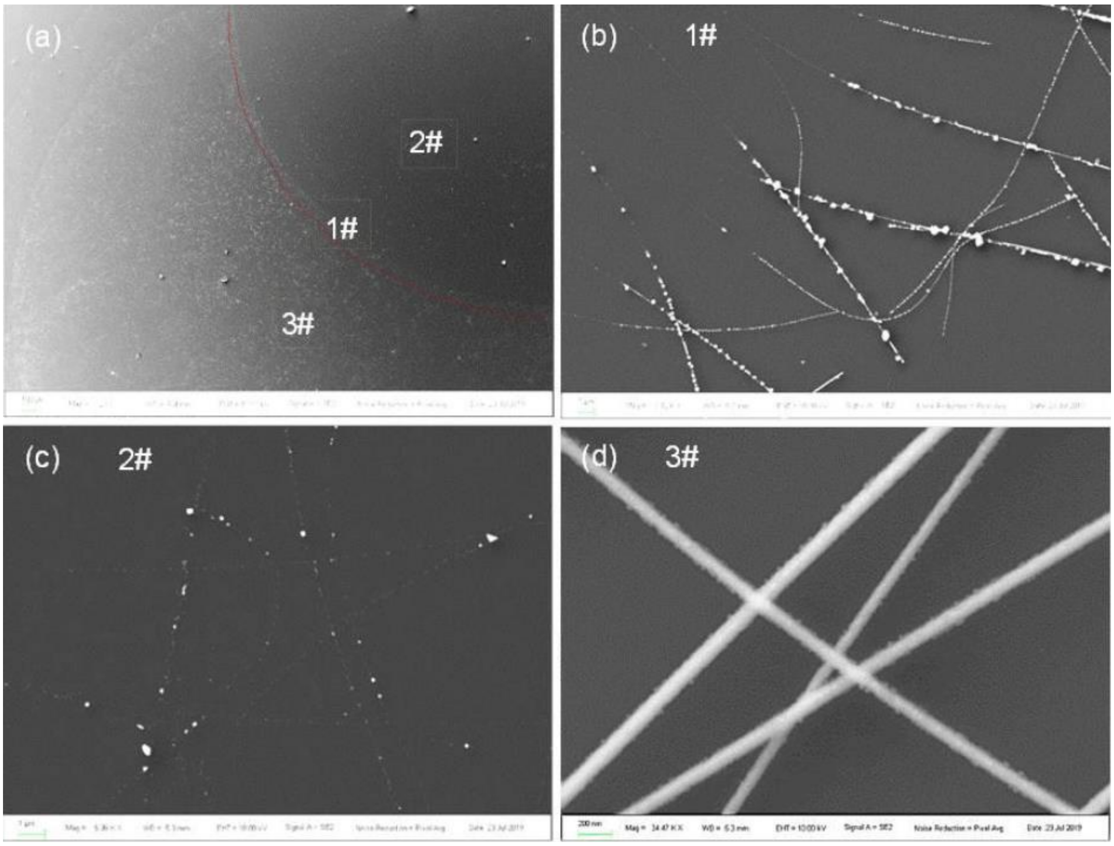

Supplementary Figure 21 | Ag NWs after H_2O_2 treatment (10%, 2 hours). (a) Low magnification SEM image of the sample after a H_2O_2 liquid was dropped where the red line is the edge of the liquid drop and the 2# region are completely immersed by H_2O_2 solution and the 3# region is outside. (b) Magnified SEM observation of the Ag nanowires in region 1#. (c) Closer observation of Ag nanowires in region 2#. (d) SEM image of Ag nanowires in region 3# with higher magnification.

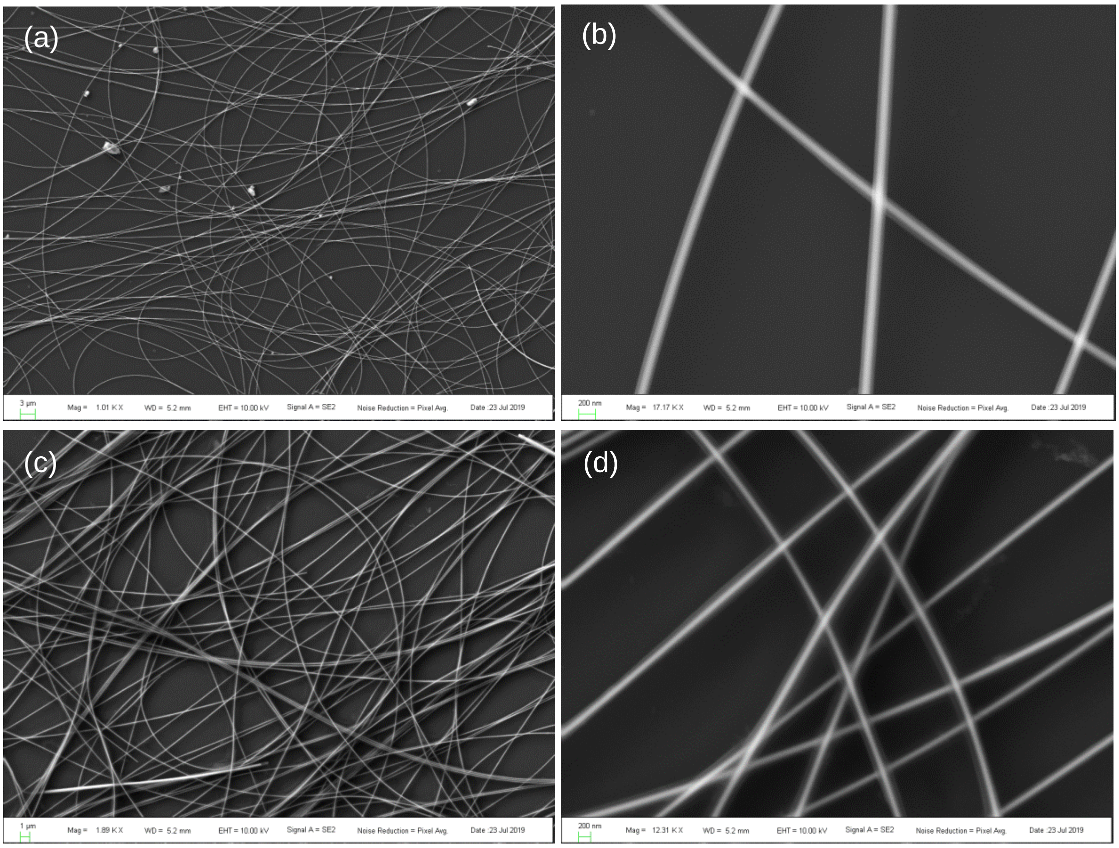

Supplementary Figure 22 | H_2O_2 solution resistance investigation. (**a-b**) The Mn_5Si_3 nanowire before H_2O_2 treatment. (**c-d**) After H_2O_2 treatment.

Supplementary Note 1 Growth mechanism of $Mn_5Si_3@SiO_2$ nanocables

The growth of high quality Mn_5Si_3 nanocables was achieved via a two-stage experiment rationally designed (Procedure A and B). Procedure A was applied to create suitable growth atmosphere condition (glassy Mn-Si-O composite rich environment) and procedure B resulted in nanowires, as shown in **Supplementary Figure 1**.

In detail, Mn and SiO powder with molar ratio of 6:1 was grinded in a mortar, until they were mixed homogeneously, which was applied as the sole reacting precursor (900mg) for experiment A. The assembly shown as Procedure: A was directly pushed into the furnace with the powder in the center of the heating zone. Then, the furnace was heated to 1250℃ in 60 minutes, which was held for 3 hours. H_2 at flow rate of 100 sccm was introduced to the chanmber until the pressure reached 20 kPa. As soon as the reaction over, the gas in the chamber was exhausted by a mechanic pump, meanwhile the furnace cooled to room-temperature with help of water coolant. At last, product of glass-like deposition region of Mn-Si-O composite formed on the ceramic sheet and also surrounding the inner wall of the protecting ceramic tube.

During the reaction in Procedure A, the mixture would firstly formed Mn_2SiO_4 (PDF# 35-0748) with excessive Mn *via* solid interaction (<1100℃), confirmed by the XRD characterization of the residue of a 60 minutes reaction at 1100℃ (**Supplementary Figure 2**). As the temperature raised to 1150℃, the precursor residue began to melt into glassy matter and serve as source to supply vaporized Mn-Si-O. Addition of excessive Mn source was helpful to avoid the decomposition of SiO, which would result in unwanted product, like SiO_2 and Si, which conversely consume Mn and prevent the formation of Mn_2SiO_4. Therefore, the key point of this stage is to make the solid reaction proceeded sufficiently as much as possible before the SiO decomposed. In this way, the main product deposited is glassy Mn-Si-O.

The basic setup of experiment B was shown as bottom panel in **Supplementary Figure 1**, where Mn-Si mixture (molar ration of 1:3, 90mg) and SiO (100mg) powders were applied as precursors. A new ceramic sheet was used. The protecting

tube was not changed, which provide the reaction with Mn-Si-O source (vaporized from the inner wall and transported to the ceramic sheet as soon as the temperature high enough). Noticeably, the molar ratio of Mn-Si was determined according to the binary Si-Mn phase diagram (**Supplementary Figure 3**), which implies that Mn_3Si display lower liquefying point and facilitate subsequent evaporation[1]. A similar heating procedure and atmosphere condition as in experiment A were applied during the reaction. High quality Mn_5Si_3@SiO_2 nanowires could be found at the marked region. When temperature high enough, Mn-Si-O composite on the inner wall of the tube would be vaporized and deposit on the ceramic sheet like in Procedure A. The formed glassy region served as matrix to incorporate active Mn-Si, Si and SiO_2 species. Mn-Si would separate out locally and nucleate as the concentration increased. Continuous supply of active Mn-Si source would then result in the longitudinal growth of nanowires. Accordingly, all of the nanowires originated from the glassy composite, as examined by careful SEM observation and EDS characterization **Supplementary Figure 4(a-d)**). Benefiting from the high quality of the nanowires, they could be uprooted mechanically from the substrate. **Supplementary Figure 5** shows the residue fracture surface as marked using brown dash circles.

As soon as the nanowire grew out from the vapor-liquid interface, they would be wrapped by SiO_2, which originates from the reaction of $SiO \rightarrow Si + SiO_2$ and $Si + MnO \rightarrow Mn + SiO_2$. Noticeably, although the dimensions of the nuclei and resulted Mn-Si crystals display a wide range, only a small part of them could evolve into ultra-long nanowires, while others failed to grow in a considerable aspect-ratio. Smooth and continuous supplement of source in the molten matrix were important.

Supplementary Note 2 The structural characterization of Mn_5Si_3@SiO_2 nanocables

The SAED pattern and HRTEM corresponding to four different regions in a long nanocable was acquired, as marked in **Supplementary Figure 8**. The upper panel of

Supplementary Figure 9(a) is the TEM image of region 1# in low magjification, and below are corresponding to SAED pattern and HETEM image. The nanocable grows along c-axis as marked. For the other three regions, the similar operation was done, as shown in **Supplementary Figure 9(b-d)**. It is obvious that the four sets of data point to the same lattice, which is to say the nanocable is in single crystal. All the nanocables exhibit the same feature.

In **Supplementary Figure 10**, it is shown that the nanocable display a very good diameter distribution, including the electrical core and the insulate shell.

Supplementary Note 3 Investigation of bendability upon a single Mn_5Si_3@SiO_2 nanocable

We employed combined bending experiments upon single nanocables and then examined their integrality using TEM. **Supplementary Figure 11**(a) shows the two nanocables chosen to be characterized (NO. 1 and NO. 2). **Supplementary Figure 11**(b) correspond to the NO. 1 nanocable in bended state with strain of 11.52% (circle diameter is 1.85um and diameter of the nanocable is 213nm). the image of it after the tungsten probe retracted was shown in **Supplementary Figure 11**(c). Afterwards, it was characterized by TEM. As in **Supplementary Figure 11**(d-i), the all parts of the nanocable segments that experienced maximum bended strain display excellent integrity, with no fracture behavior detected. The operation upon the other nanocable was recorded in **Supplementary Figure 12**(a-c). **Supplementary Figure 12**(d) shows its recovered configuration. Considering that the maximally bended region locates on the Si_3N_4 film, we cut it off mechanically and then moved the targeted segment to the carved position using a tungsten probe (**Supplementary Figure 12**(e-f)). **Supplementary Figure 12**(g-i) correspond to the TEM examination of the bended region, which indicates that the nanocable can withstand the bended strain of 13.02% without fracture.

Supplementary Note 4 The electrical performance of a single $Mn_5Si_3@SiO_2$ nanocable largely bended

As in **Supplementary Figure 14**, a nanocable with moderate length was firstly transferred onto an Au pattern fabricated via UV-lithography and lift-off process on Si_3N_4 (180nm)/Si substrate. After removing SiO_2 shell with focused Ga^+ beam, one end of the nanocable was fixed onto the tip of tungsten probe via electron beam assisted Pt/C deposition, and the other end was fixed onto the Au pattern (**Supplementary Figure 14**(b)). The tungsten probe could move freely in the chamber space, creating random bending deformation of the nanocable. During the *I-V* measurement, another tungsten probe contacted the Au pattern (**Supplementary Figure 14**(b)).

I-V curves of the nanocable corresponding to various bending deformations (**Supplementary Figure 14**(b-f)) were acquired, as shown in **Supplementary Figure 14**(h)**.** In (b), the nanocable was simply folded, while in (c) it was bended into a more complex configuration. Then, it was stretched again into a straight configuration (d-f). For all the configurations, the *I-V* characteristics were acquired, as shown in **Supplementary Figure 14**(h). It is obvious that the electrical property displays no degeneration during the repeated bended process. Noticeably, in (d), the end close to the tungsten tip was bended into a circle, whose radius decreased as the nanocable was stretched forward, until the circle almost wrapped the tip, as in (f-g). These operations indicate that the nanocable exhibits robust electrical property even under large bending deformation, without structural fracture and distinguished electrical performance degeneration.

Supplementary Note 5 The electrical performance and structural change of $Mn_5Si_3@SiO_2$ nanocables at elevated temperatures

In order to investigate the temperature dependent resistance of a single nanocable, a heating panel was used to raise the temperature. A thermal imager was applied to check the precise device temperature,as shown in **Supplementary Figure 16**. The emissivity of the substrate was firstly calibrated using Al_2O_3 sheet and plastic as references, whose value are ~0.92. As in **Supplementary Figure 16(b)**, they display almost the same surface temperature distribution, implying the same emissivity value. Then, the exact temperatures of the substrate were determined as the hot plate heated. At the meanwhile, resistances were recorded.

Supplementary Figure 17 shows the change of Au electrodes after the heating process. It is obvious that some degree of melting behavior occurred. Therefore, a higher temperature is not applicable to this experiment, although the nanocable can withstand a much higher temperature.

Supplementary Figure 18(a-b) shows the SEM images of the nanocables after annealing procedure at 500℃ for 2 hours. It is obvious that no visible change found. In **Supplementary Figure 18(e-f)**, SEM images of the nanocables after annealing process at 600℃ for 2 hours are shown. Noticeably, fatal change began to occur, *i.e.* the Mn_5Si_3 core degenerated followed by Mn-O composite separate out. In **Supplementary Figure 18(d)**, one can see the lacking segment of Mn_5Si_3 core and formed composite outside the surface. To check the availability of their electrical performance, a single nanocable was made into double electrodes device, as in **Supplementary Figure 18(e).** I-V characteristic implies that electrical property is robust without degeneration.

Supplementary References

[1] Okamoto, H. Mn-Si (Manganese-Silicon), JPE 12, 505-507 (1991).
